# Nuclear Protein Aggregates Disrupt RNA Processing and Alter Biomechanics in a Muscle Cell Model of OPMD

**DOI:** 10.14336/AD.2025.0699

**Published:** 2026-08-18

**Authors:** Milad Shademan, Sarah Flannery, Erik Bos, Tom M.J Evers, Vahid Sheikhhassani, Alireza Mashaghi, Benno Kusters, Baziel van Engelen, Thomas H Sharp, Roman Fischer, Benedikt M Kessler, Vered Raz

**Affiliations:** ^1^Leiden University Medical Centre, Leiden, Leiden, The Netherlands.; ^2^Target Discovery Institute, Centre for Medicines Discovery, Nuffield Department of Medicine, University of Oxford, Oxford UK.; ^3^Medical Systems Biophysics and Bioengineering Laboratory, Leiden Academic Centre for Drug Research, Leiden University, Leiden, the Netherlands.; ^4^Radboud University Medical Center, Nijmegen, The Netherlands.; ^5^School of Biochemistry, Bristol University, Bristol, UK

**Keywords:** Protein aggregates, Pathological Protein, PABPN1, RNA metabolism

## Abstract

Aggregation of RNA-binding proteins (RBPs) is a hallmark of several age-related neuromuscular diseases. However, our understanding of how these aggregates drive dysfunction is often limited by the use of non-disease-relevant models. Oculopharyngeal muscular dystrophy (OPMD) is caused by a short alanine expansion mutation in the PABPN1 gene, which leads to nuclear aggregation of the protein. To investigate how these aggregates impair muscle cell function, we developed a muscle cell model with inducible expression of the pathogenic PABPN1 (A16) variant and confirmed its relevance to OPMD. Using subcellular fractionation combined with mass spectrometry and RNA sequencing, we examined the molecular consequences of nuclear PABPN1 aggregation. In the cytoplasmic fraction, we observed significant impairments in cellular metabolism and biomechanics. In the nuclear fraction, RNA metabolism was broadly disrupted, and additional RBPs were significantly enriched in insoluble aggregates. Importantly, mRNAs trapped within the aggregates were associated with impaired nuclear export and decreased translation efficiency, and the pathogenic PABPN1 variant led to reduced endogenous PABPN1 levels. Our findings support a model in which OPMD pathology arises from reduced levels of soluble PABPN1 due to nuclear aggregation and establish a mechanistic link between RBP aggregation and muscle cell dysfunction, highlighting shared pathological pathways across neuromuscular and neurodegenerative diseases.

## INTRODUCTION

RNA-binding proteins (RBPs) play an indispensable role in RNA-dependent cellular processes that shape the cellular proteome [1-3]. Versatile ribonucleoprotein complexes regulate RNA metabolic processes, from synthesis to translation, in both nuclear and cytosolic compartments [4, 5]. Dysfunctions of RBPs are associated with many human diseases, with a particularly high prevalence in age-associated neurological and neuromuscular disorders (NMDs). Tissue-specific limited expression levels have also been implicated in the aging of neuromuscular tissues [6, 7]. Heritable mutations in RBPs cause neuromuscular degenerative diseases, some of which involve aggregation-prone proteins or proteins that modify aggregates [8, 9]. Cytosolic protein aggregates are formed by RBPs, such as FUS and HNRNPA1, which are associated with several neuromuscular degenerative (NMD) diseases, including amyotrophic lateral sclerosis (ALS) and frontotemporal dementia (FTD) [10-12]. Additionally, RBP aggregates can be nuclear, as with those involving PABPN1, which causes oculopharyngeal muscular dystrophy (OPMD) [13].

OPMD is a rare autosomal dominant disorder with a prevalence of 1 in 100,000, characterized by progressive muscle weakness beginning in midlife [14]. Normal PABPN1 contains an alanine stretch (10 alanine residues) at the N-terminus of the protein. A short expansion of the alanine stretch (+1 to +8) causes OPMD. The expanded PABPN1 forms nuclear aggregates that are associated with OPMD, though not with disease severity [15, 16]. Although PABPN1 is expressed throughout the body, OPMD symptoms primarily affect skeletal muscle, suggesting muscle-specific disease mechanisms. The factors involved in PABPN1 aggregation and modulation of muscle cellular dysfunction remain largely unknown.

PABPN1 plays a key role in mRNA processing, including regulating poly(A) tail length and alternative polyadenylation (APA) [17]. In disease models with reduced PABPN1 expression levels or PABPN1 aggregates, APA occurs genome-wide [18]. PABPN1 primarily influences alternative polyadenylation (APA) at the 3'-untranslated region (UTR) of transcripts, favoring a distal-to-proximal shift that produces shorter, more stable transcripts [19]. Additionally, PABPN1 is involved in the nuclear export of mRNA [20, 21]. Although PABPN1 predominantly functions in the nucleus, like other RBPs, it shuttles to the cytoplasm. However, the role of PABPN1 in the cytoplasm is not well understood, as is the mechanism by which nuclear aggregates cause cellular dysfunction.

In this study, we investigated the effect of expanded PABPN1 insoluble nuclear aggregates on muscle cell function using an inducible cell model that allowed us to overcome the cytotoxic effect typically associated with PABPN1 aggregates. We analyzed the cell proteome and transcriptome in nuclear, cytoplasmic, and insoluble subcellular fractions, validating the protein networks affected by PABPN1 aggregates. Our study provides insights into the molecular mechanisms by which nuclear aggregates lead to cellular dysfunction.

## MATERIALS AND METHODS

### PABPN1 constructs and lentivirus production

The expanded PABPN1 (Ala16) transgene was cloned into the pCW57-MCS1-2A-MCS2 doxycycline (Dox) inducible lentiviral vector (Addgene plasmid #71782). Subsequently, the FLAG tag was also fused to the C-terminus of the PABPN1 sequence. FLAG was added using the QuikChange Site-Directed Mutagenesis Kit (Agilent technology #200519), according to the manufacturer protocol and with the following primer set: Forward: gactacaaggatgacgatgacaagTAAATTAGGAGGAGAGAGAGG; Reverse: TTActtgtcatcgtcatccttgtagtc GTAAGGGGAATACCATGATG. The cloning was confirmed by Sanger sequencing. Lentivirus production was performed as detailed in [22].

### Cell culture

The immortalized human muscle clone (MB135) was generated from primary human muscle cells, collected from a healthy female donor, and were previously described in [23, 24]. Growth conditions were as described in [25]. In brief, myoblasts were cultured in growth medium (F10 (Gibco) medium supplemented with 15% FCS, 1 ng/ml bFGF, 10 ng/ml EGF and 0.4 µg/ml Dexamethasone). Cells were passaged when reaching confluence 50-80%, and cell cultures were renewed every 6-8 weeks. Cell cultures did not reach 100% confluence to avoid spontaneous differentiation. Muscle cell differentiation was done at high confluency (85-95%) in DMEM+2% horse serum for 3-5 days. Stable cells containing the inducible Ala16 (with or with Flag tag) were made by incubation with lentivirus particles + polybrene as detailed in [22]. Stable cells were selected with puromycin (2.5 μg/ml). The Ala16 transgene was induced with 4 μg/mL doxycycline hydrochloride (D5207, Sigma Aldrich), and DMSO was used for (uninduced) vehicle-treated cells. For high content screening (HCS), cells were seeded on a Nunc 96 well plate; for live cell confocal microscopy, cells were seeded in a µ-Slide 8 Well high ibiTreat (80806, IBIDI) slide. For electron microscopy, cells were seeded in µ-Dish 35 mm, high Grid-500 ibiTreat (81166, IBIDI) dishes. For experiments in proliferating cells, cells were seeded at 40% confluence (20,000 cells per well), and for differentiation cells were seeded at 90% confluence (50,000 cells per well).

### Subcellular fractionation

For subcellular fractionation, cells were cultured in a 6-well plate (~3 million cells per well). Cells were harvested by trypsinization, and a quick spin-down at 4°C. Cell pellets were incubated on ice for 30 minutes with the cytosolic lysis buffer (150mM NaCl, 50mM HEPES (pH 8), 1 mM DTT, and protease inhibitor cocktail (Roche)). The cytosolic supernatant is collected after centrifugation at 3500g, 4°C, for 10 minutes. The remaining pellet was washed with excess PBS and, after additional centrifugation, solubilized in a nuclear lysis buffer (150mM NaCl, 50mM HEPES (pH 8), 0.5% w:v Sodium deoxycholate, 0.1% Triton, 1 mM DTT, and protease inhibitor cocktail). After 15 minutes of incubation on ice, the nuclear supernatant fraction was collected after centrifugation at 5000g, 4°C for 10 minutes. The remaining pellet contained the insoluble fraction and was solubilized in PureLink (Invitrogen #12183018A) for RNA extraction, or in 2% SDS for protein extraction. Protein concentration from cytosolic and nuclear fractions was determined using the protein assay kit (BioRad #5000001).

### Protein extraction and western blot analysis

Protein extraction of the soluble fraction was made with a lysis buffer (20 mM Tris, pH 7.4, 150 mM NaCl, 5 mM EDTA, 0.1% NP40, and 1 mM DTT and 1x protease inhibitor cocktail). After sonication and centrifugation (1 min, 13000g, at 4°C), the supernatant containing the soluble proteins was transferred to a new tube, and the pellet, containing the insoluble proteins, was washed once in PBS, dissolved in loading buffer, sonicated and spin down before heat inactivation. Insoluble proteins were extracted from the pellet with lysis buffer +2% SDS. The protein amount was determined in the soluble fraction and the proportional aliquots from the paired insoluble fraction for equal loading on SDS-PAGE. Protein aliquots were separated on 10% SDS-PAGE. Western blotting was carried out with a PVDF membrane. Bulk proteins were visualized with the No-Stain Protein Labeling Reagent (#A44717, ThermoFisher) and imaged using the iBright Imaging System (ThermoFisher). The membrane was blocked with 5% dried milk powder (T145.2, Carl Roth). Primary antibody incubation was carried out at 4°C overnight, and secondary antibody incubation at room temperature for one hour. Antibodies are listed in Supplementary Table 1; the antibody for PABPN1 is described in [26]. An Odyssey CLx Infrared imaging system (LiCOR, NE. USA) was used to detect the fluorescent signal. Quantification of protein abundance was done using ImageJ. Values were corrected for background and normalized to loading controls. Western blot quantification was carried out with ImageJ. Normalization was made for both the No-Stain and housekeeping signal. Full western blot images are provided in Supplementary Figure 1.

### Mass spectrometry and data analysis

Sample aliquots containing 50 µg protein were incubated with 250 Units of Benzonase® Nuclease (Sigma-Aldrich) for 10 minutes at room temperature, then solubilized in 5% SDS. Cysteine residue reduction and alkylation were performed using 10 mM tris(2-carboxyethyl) phosphine and 50 mM iodoacetamide at room temperature for 30 min. Subsequent tryptic digestion was performed by S-trap micro (ProtiFi) according to the manufacturer’s instructions. After elution, peptides were dried by vacuum centrifugation and stored at -20 °C for MS analysis.

Peptide samples were reconstituted in 3% acetonitrile 0.1% formic acid before MS analysis, then 200 ng were analysed by liquid chromatography-tandem mass spectrometry (LC-MS/MS) using a Dionex Ultimate 3000 UPLC coupled to a Q-Exactive mass spectrometer (ThermoFisher Scientific). Peptides were trapped on an Acclaim™ PepMap™ 100 C18 HPLC Column (PepMapC18; 300 µm x 5 mm, 5 µm particle size, Thermo Fischer) using solvent A (0.1% Formic Acid in water) at a pressure of 60 bar, and separated on an Easy Spray PepMap RSLC column (75 µm i.d. x 2 µm x 50 mm, 100 Å, ThermoFisher) at 250 nL/min over a 60 min gradient from 5% to 35% acetonitrile in 5% DMSO, 0.1% formic acid. MS data was acquired in data-independent acquisition (DIA) mode, with full scan MS spectra acquired in the Orbitrap, from 20 m/z windows over a scan range of 495 to 995 m/z, with an overlap of +/- 2 Daltons, resolution 35000, AGC target 3e6, maximum injection time 55 ms and fragmentation at 28% normalized collision energy. MS/MS spectra were also acquired with a resolution of 17500, AGC target 1e6. Raw MS data were searched (UniProtKB reviewed proteome database UP000005640) using DIA-NN v1.8 in library-free mode with automatic mass accuracy optimization, cross-run normalization enabled, 1 missed cleavage permitted, fixed cysteine carbamidomethylation, and methionine oxidation as a variable modification. DIA-NN output data, containing 6604 annotated proteins, were log2 transformed. For fractionation validation, protein abundance was normalized by centering the median per fraction (cytosolic soluble (C), nuclear soluble (N), and insoluble fraction (Ins) from Dox-induced and vehicle-treated cell cultures with three biological replicates per fraction per genotype). Fractionation efficiency (C vs. N or N vs. Ins.) was assessed by paired differential analysis by two-sample t-test in Perseus Software v2.0.7.0. Ala16 PABPN1 effect per fraction was tested with Dox-induced vs. vehicle-treated samples, with the following inclusion criteria: 1. minimum read >=1 per sample, 2. minimum read >=67% (2/3) per group (N=3). Protein abundance was normalized by median centering per fraction. Differential analysis was performed by two-sample t-test in Perseus Software v2.0.7.0. The expression profiles between fractions were determined by hierarchical clustering with Euclidean distance using Z-scores in Perseus Software v2.0.7.0. Proteomic differential analysis is found in Supplementary Figure 3. The correlation between PABPN1 levels and other protein levels was calculated with Pearson correlation in GraphPad Prism 9.3.1.

### RNA extraction, library preparation, RNA sequencing, and analysis

RNA was extracted from cytosolic, nuclear, and insoluble fractions using the PureLink RNA Mini Kit (Invitrogen 12183018A), according to the manufacturer protocol, continue with on-column DNase treatment PureLink™ DNase Set (Invitrogen 12185010). RNAs were stored at -80. RNA integrity was quantified using Qubit and checked on an RNA 6000 Nano Agilent Lab-on-a-Chip kit with Bioanalyzer Systems before cDNA library preparation. One hundred nanograms of RNA per sample were used to prepare poly(A) libraries with a single PCR cycle and 3'-UTR enrichment. This approach captures 3'-UTR changes that closely reflect cellular conditions, providing an accurate snapshot of mRNA dynamics in situ. The library preparation protocol and RNA sequencing are detailed in [27].

All reads in FASTQ format were first filtered using *Cutadapt* (v2.10), removing all remaining adapter sequences. *MultiQC* program in Python was used for quality control (QC) assessment of FASTQ files. The remaining reads were aligned to the Ensembl transcriptome version 104 using STAR (v2.7.5a), including UMI-based deduplication using UMI-Tools (v1.1.1), generating a transcriptome-based alignment in BAM format. We used the same Ensembl transcript annotation version 104 to create a customized transcript annotation GTF file for all Ensembl annotated transcripts. With the annotation and human transcriptome-based alignment files as input, we quantified the reads at the coding regions using *featureCounts* (v2.0.1). For downstream the nuclear and cytosolic mRNA samples were normalized together (N=12 samples), while the insoluble samples were normalized separately (N=6 samples). All analyses were performed using RStudio Software RStudio 2022.02.3 (Build 492) using R Statistical Software (v4.2.3).

The APA-shift calculation was made as described in [27] on raw reads counts after exclusion criteria. The ratio between proximal to distal was calculated in R (version 4.3.1) using the equation [log(1+Proximal) - log(1+Distal)], and the APA-shift was calculated between induced and uninduced conditions. The APA-shift calculation was done on the cytosolic and nuclear fractions and therefore under the hypothesis that the APA-shift does not differ between fractions. APA-shift was fist calculated for both fractions together (N=6 per fraction; exclusion criteria: >12 sum of the reads/3-'UTR of a transcript, in average >1 per sample), and subsequently, the significant APA-shift transcripts were sorted per fraction (cytosolic or nuclear), and p-values were recalculated per fraction. APA-shift calculation per fraction (N=3 per fraction; exclusion criteria: >6 sum of the reads/3-'UTR of a transcript, in average >1 per sample) was made for each fraction separately. The APA-shift is in log2, APA-shift >0 indicates a shift to proximal, and <0 suggests a shift to distal. APA-shift significance per fraction was calculated with Student’s *t*-test, corrected for FDR.

Transcript differential expression analysis was made in edgeR Bioconductor package (v3.42.4) [28]. Transcript list excluded read count =<1 count per sample. TMM normalized read counts were log-transformed. Main variations between samples were assessed unsupervised with the principal component analysis (PCA). Differential expression analysis was calculated with the Empirical Bayes in edgeR with the *decideTest* and *p*-value<0.05 corrected for false discovery rate (FDR). Differential expression analysis was made between fractions (N=6 per fraction) or between Dox-induced and vehicle-treated cells (N=3 per fraction). Volcano plot visualization of differential expression was made in https://ggvolcanor.erc.monash.edu/ [29]. Expression profiles across fractions was determined in Perseus Software v2.0.7.0. Hierarchical clustering of Z-scores was made for the 877 overlapping DE transcripts between cytosolic and nuclear fractions using Euclidean distance.

### RT-qPCR

RT-qPCR was conducted on RNA from the Dox-induced and vehicle-treated cells. RNA was extracted with the PureLink RNA Mini Kit (Invitrogen 12183018A). 500 ng RNA was reverse transcribed for cDNA synthesis using the QuantiTect Reverse Transcription Kit (QIAGEN) and random primers, following the manufacturer’s instructions. Subsequently, qPCR amplification was performed with the QuantiNova SYBR Green kit (QIAGEN) using 5 ng RNA, with technical duplicates, using a standard amplification protocol at a melting temperature of 60°C. The PCR detection was carried out in the CFX384 system (BioRad). Samples with CT values above 35 were excluded from the analysis to eliminate potential noise. The average CT values from the technical duplicates and normalization to the HPRT1 gene were used for ddCT calculation. Two primer sets were used: a primer set to exon 3-4 PABPN1 (ENSG00000100836), amplifies both the Ala16-PABPN1 transgene and the endogenous PABPN1, and the second primer set to the 3’-UTR amplifies only the endogenous transcript. Primer sets were designed with the NCBI Primer design tool (www.ncbi.nlm.nih.gov/tools/primer-blast), and the primers are listed in Supplementary Table 2.

### Immunofluorescence and staining

For immunofluorescence cells were cultured in plastic bottom 96-well plates (ThermoFisher: Nunc™ MicroWell™ 96-Well Microplates #260860; 96 Well Black/Clear Bottom Plate #165305 for HCS platform) or in IBIDI plates (confocal imaging). Cell fixation was performed with 4% Formaldehyde in PBS for 5 minutes. KCl treatment was before to cell fixation was with 1M KCl for 15 minutes, followed by fixation with 4% Formaldehyde for 5 minutes. Subsequently, permeabilization was performed with 1% Triton-X100 for 10 minutes, followed by 1X PBS washing and first antibody incubation for one hour at room temperature. The first antibody was washed 3-times with excess PBT for five minutes each. Incubation with a fluorophore-conjugated secondary antibody and DAPI was carried out for 30 minutes, followed by PBT washing (3-timess 5 minutes each). Antibodies are listed in Supplementary Table 1. Antibody incubation and washing steps were made with PBS+0.05%-Triton-X (PBT). Cells were left in PBS during imaging.

### Cellular assays

Cellular assays were conducted in vehicle-treated or Dox-induced cell cultures for 4 days.

*Fusion index:* differentiated cells were marked with the MF20 antibody, staining the myosin heavy chain isoforms (MyHC) [30]. Fusion index was calculated from the proportion of nuclei in MyHC objects. Fusion index is a calculated value which is measured in muscle cell culture cultivated under differentiated conditions, the value reports muscle differentiation.

*Oligo-dT in situ hybridization to detect bulk poly(A) RNA*: cells were cultured in 96 Well Black/Clear Bottom Plate (ThermoFisher #165305), and were fixed using 3.7% formaldehyde for 15 minutes at RT. After two PBS washes, the cells were incubated in protease III and diluted 1:30 in PBS (#322337 Advanced Cell Diagnostics) for 15 minutes at RT. After twice PBS washes cells were incubated in a hybridization buffer (#10369 Cepham Life Sciences) for 15 minutes at RT. Incubation with 5’-Cy5-Oligo-dT12-18 probe (#26-4400-02 Gene Link), diluted 1:1000 in hybridization buffer, was carried out overnight at 40°C in a humidified chamber. The following day, washes were carried out at 40°C for 5 minutes with 4x, 2x, and 1x SSC buffer and with PBS. Finally, the cells were incubated with Hoechst and kept in PBS during imaging.

*RNAscope:* Spatial localization of a single RNA molecule was carried out in adherent muscle cells cultured in 96 Well Black/Clear Bottom Plate (ThermoFisher #165305), as detailed in [31]. Fluorescent Multiplex Assays kit according to the manufacturer’s protocol (ACD biotech) with the following modifications: the protease solution was diluted 1:30, and the amplifier solutions were diluted 1:2. Genes included in the RNAscope: The positive control probe mix, provided by the company (POLR2A, UBC, and PPIB). The second probe mix included PABPN1 and MYF5. The probes for POLR2A, UBC, PPIB, and MYF5 are from the Human probe set (ACD biotech). The PABPN1 probe is from the mouse set, and the homology between humans and mice is >95% for the probe regions.

*LMB treatment:* cells were treated with 37.5 nM LMB (LKT Labs, St. Paul, USA) for 3 hours at 37°C or DMSO (dilution 1:1000) as Mock control.

*Protein synthesis assay:* the protein synthesis assay kit (Cayman Chemicals #601100) was conducted according to the manufacturer protocol using azido-O-propargyl-puromycin (OPP)-488 (named here OPP). A 30-minute pre-incubation with 20µM cycloheximide was used as a negative control. Hoechst was added after fixation. OPP was imaged with a 488 filter.

*Mitochondrial activity:* JC-1 staining was carried out in living cells with JC-1 5µM (final concentration), and Hoechst 1mM final concentration (33342 ThermoFisher) added to the growth medium and incubated for 30 minutes at 37°C. Cells were washed once with PBS and kept in a growth medium during imaging. JC-1 was imaged with 488 and 560nm filters.

*Glucose uptake assay:* was conducted in cell cultures treated with Dox and incubated in a differentiation medium for four days. According to the manufacturer protocol, the glucose uptake was determined with the Glucose Uptake-Glo™ (Promega # J1341). Fluorescence was measured with the SpectraMax iD3 multi-mode microplate reader (Molecular Devices) recorded at 1-second integration.

### Imaging platforms and image quantification

The CellInsight CX7 LZR high-content screening (HCS) platform was used for high-content imaging. The accompanying HCS Platform spot detector and co-localization toolbox (ThermoFisher Scientific) performed a cell-based analysis.

Imaging was used to calculate the differentiation index, which was done with a 10x objective covering over 12,000 nuclei per well, using the co-localization toolbox.

Imaging for PABPN1 quantification, oligo-dT, OPP, and JC1 was made with a 20x objective, covering at least 5000 nuclei per well. The spot detection toolbox was employed. JC-1, OPP, and RNAscope were analysed from the perinuclear region. Oligo-dT-Cy5 and PABPN1 signals were measured from both nuclear areas.

Confocal microscopy imaging: Fixed single nuclei were imaged with a Leica DMi8 with the Andor Dragonfly spinning disc module using a 40x/1.3 or 63x/1.3 oil immersion objective. Identical imaging settings, including exposure time, laser power, the excitation-emission range, and Z-stacks step size, were employed within an experiment. Quantifications of confocal images were carried out in ImageJ.

### Electron microscopy

The EM embedded OPMD muscle biopsy was reported in [32]. Differentiated cell cultures were fixed in 1.5% glutaraldehyde in 0.1 M Sodium Cacodylate buffer for 2 hours and successively incubated in 1% Osmium Tetroxide in 0.1 M cacodylate buffer for 1 hour and in 1% Uranyl Acetate in water for 1 hour. The cells were then dehydrated through a series of incubations in Ethanol (70-100%) for 90 minutes and embedded in Epon. The flat embedded cells were sectioned with an ultramicrotome (UC6, Leica, Vienna) using a 35-degree diamond knife (Diatome, Biel, Switzerland) at a nominal section thickness of 90 nm. The sections were transferred to a formvar, and a carbon-coated 1 ×2 mm copper slot grid and then stained for 20 minutes with 7% uranyl acetate in water for 10 minutes with lead citrate. EM images were recorded using a Tecnai 12 electron microscope (Thermo Fisher Scientific) with an EAGLE 4k×4k digital camera.

### Single-cell biomechanics

Cell membrane force experiments were performed using a CellHesion 200 instrument (JPK, Berlin, Germany) equipped with a Petri dish heater to maintain a temperature of 37°C. Cantilevers with a cylindrical tip having a 5-micron end radius and nominal spring constants ranging from 0.166 - 0.179 N m-1 were used (Bruker, SAA-SPH-5UM). The spring constant calibration was performed using the thermal noise method [33]. Cells were approached at a loading rate of 5 µm s-1 with a maximum force set-point of 0.473 nN. Each cell was indented three times, and 10 cells were probed per condition. Between cell indentations, the substrate was probed to ensure the tip’s cleanliness. Cells were indented above the nuclear region to reduce variability and substrate artifacts. All experiments were analysed using JPK Data Processing to determine the Young’s Modulus of the cells. The Hertz model was used for calculating Young’s modulus, as described in [34, 35]. This model is valid for small indentation depths and is expressed as follows for a spherical indenter:

$$F=\frac{4\sqrt{R}}{3}\frac{E}{1-\nu^{2}}d^{3/2}$$

where *F* is the indentation force, *R* is the radius of the indenter, *E* is Young’s modulus, *ν* is Poisson’s ratio and *d* is the indentation depth.

### Statistical and bioinformatics analyses

Differential expression analysis of poly(A) RNA was conducted in R 4.3.1 (Limma-Voom pipeline +EdgeR), and of proteomics in Perseus software v2.0.7.0. Test were corrected for FDR (Benjamini-Hochberg method). Statistical tests for cellular and molecular assays (N=3 or 4 biological replicates) were made in GraphPad Prism 9.3.1 using a non-parametric test. The specific test is indicated in legend to Figure.

Enrichment analysis for the differential abundance proteins in each cluster and DE transcripts in cytosolic fraction was conducted within DAVID (v2023q3, https://david.ncifcrf.gov). The gene network databases Reactome and Gene Ontology were selected for enrichment analysis. Fifty proteins were enriched in the Metabolism of RNA based on the Reactome database and were used for the Gene-Disease enrichment analysis at https://maayanlab.cloud/Enrichr [36].

The RBP protein list, RBP2GO, is from https://rbp2go.dkfz.de [37]. The muscle protein list was downloaded from https://amigo.geneontology.org/amigo using the filters: muscle, human, protein (N=1371 proteins).

Reads at the 3′-UTR were visualized using IGV version 2.16.0. Candidates with 3′-UTR lengths above 1 kb were selected for visualization.

Linear regression analysis for transcriptome-proteome correlation was made in www.statskingdom.com.

### Ethics

OPMD muscle biopsy collection has been approved by the Radboud Medical center ethics committee in accordance with the ethical standards laid down by the 1964 Declaration of Helsinki. The patients signed on informed consent prior to biopsy collection.

## RESULTS

### The A16 muscle cell model represents PABPN1 aggregation in OPMD

**Figure 1.**
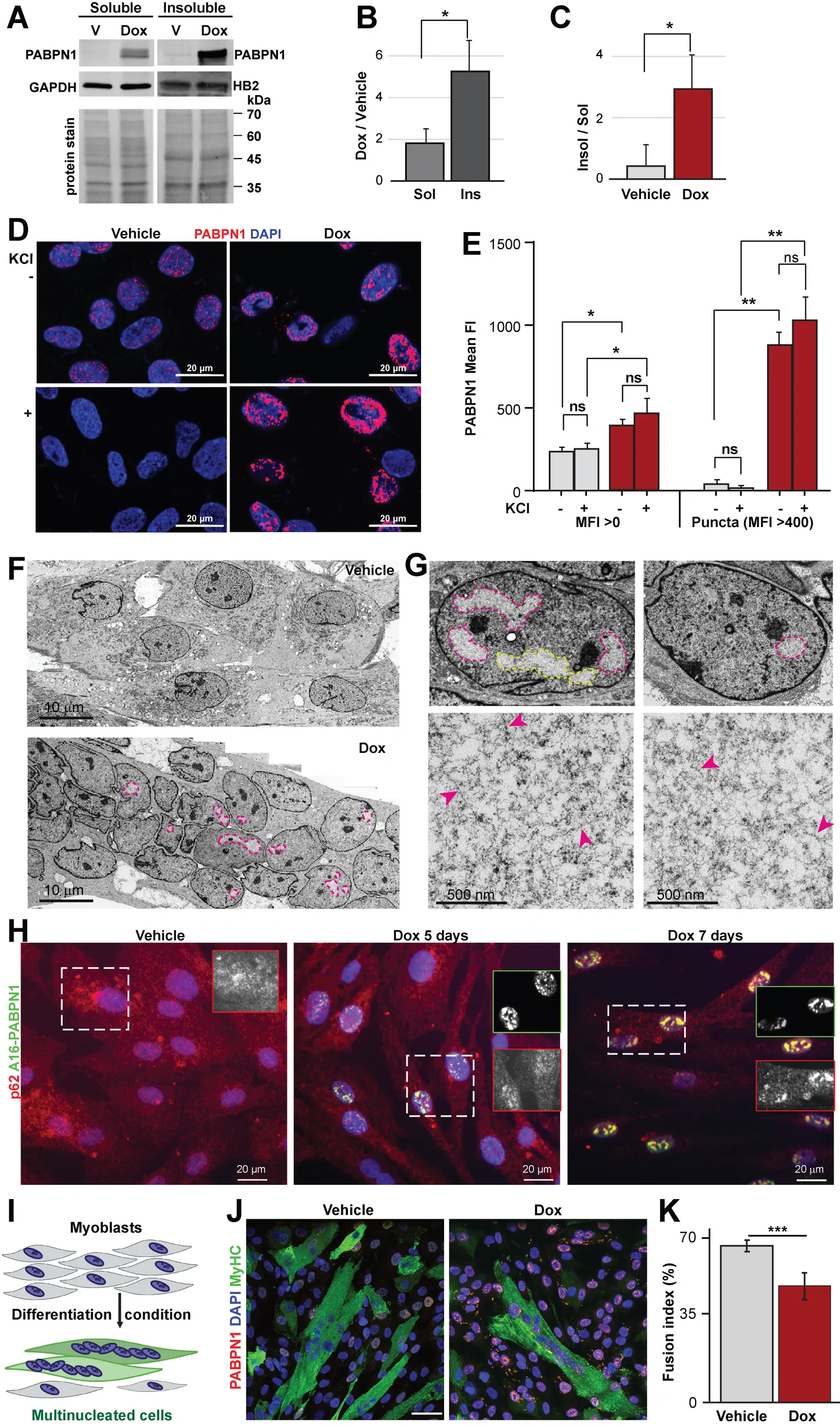
**Inducible A16-PABPN1 forms insoluble aggregates in muscle cells**. (**A**) Western blot analysis of PABPN1 abundance in soluble and insoluble fractions in vehicle-treated (V) or Dox-induced cell cultures. GAPDH labels the soluble fraction and HistoneB2 labels the insoluble fraction. No staining is a loading control. Molecular weight in kDa is depicted. (**B**) Bar graphs show PABPN1 fold change (FC) between Dox-induced and vehicle-treated in the soluble and insoluble fractions. (**C**) Bar graphs show PABPN1 FC between soluble and insoluble fractions in vehicle-treated or Dox-induced cell cultures. Means and standard deviations for B and C are from N=4 biological replicates. (**D**) Confocal images of PABPN1 (red) immunofluorescence in vehicle-treated and Dox-induced cell cultures under proliferating conditions +/- KCl treatment. The scale bar is 20 μm. (**E**) Bar graphs show mean nuclear PABPN1 fluorescence intensity with thresholding in a vehicle-treated or Dox-induced cell culture +/- KCl treatment. Mean and standard deviation are from N=3 biological replicates. Two-way ANOVA was used to determine statistical significance; p<0.05 is denoted with *, and p<0.01 is denoted with **. (**F**) A stitched electron microscopy (EM) image of the vehicle-treated or Dox-induced fused cell. The dashed lines encircle EM fluorescent regions. The scale bar is 10 μm. (**G**) Examples of single myonuclei in Dox-induced fused cells with large (left) and small (right) EM lucent regions and magnification of the lucent region (bottom). Arrowheads indicate fibrils. The scale bar is 500nm. (**H**) Representative immunofluorescence overlay images of p62-Cy5 (red) and A16-FLAG-Alex488 (green) in vehicle-treated and Dox-induced cell cultures for 5 or 7 days. Inserts of p62 (gated in red) or A16-FLAG (gated in green) are from the gated area (dashed white line). The scale bar is 20 μm. (**I**) Schematic representation of myoblasts cell differentiation. Differentiated cells are depicted in green. (**J**) Images of PABPN1 (red) and MyHC (green) immunofluorescence in vehicle-treated and Dox-induced cell cultures for 5 days. The scale bar is 40 μm. (**K**) Bar graph of the percentage of differentiation (fusion index) in Dox-induced and vehicle-treated cells. Means and standard deviations are from N=4 biological replicates. One-way ANOVA was used to determine statistical significance; p<0.005 is denoted with ***.

To explore the molecular mechanisms driven by PABPN1 nuclear aggregates, we generated stable cells that express an A16 expansion (16 alanine repeats) in the PABPN1 cDNA under a tetracycline-inducible promoter. We investigated the +6 alanine expansion because it is the most prevalent mutation among Dutch patients with OPMD [16]. Inducible expressions overcame the toxic effects of constitutive expanded PABPN1 expression [38, 39]. Western blot analysis confirmed A16-PABPN1 expression after doxycycline (Dox) treatment (Fig. 1A). The accumulation of soluble PABPN1 after Dox induction was twice as high as in vehicle-treated cells (Fig. 1B). The accumulation of insoluble PABPN1 in Dox-treated cells was six times higher than in vehicle-treated cells (Fig. 1B). The level of insoluble PABPN1 was three times higher than that of soluble PABPN1 (Fig. 1C). Thus, A16 PABPN1 overexpression leads to higher insoluble PABPN1 accumulation than soluble PABPN1 accumulation. We refer to the Dox-induced cell culture as A16.

Immunofluorescence analysis of Dox-treated cells after KCl treatment confirmed the presence of insoluble PABPN1 in Ala16 cells (Fig. 1D). KCl removes the signal from soluble PABPN1, leaving aggregated PABPN1 intact [40]. In A16 cells, the mean fluorescence intensity (MFI) of total PABPN1 increased 1.5- to 2-fold compared to vehicle-treated cells (Fig. 1E, MFI > 0), which is consistent with the results of the Western blot. Aggregated PABPN1 can be identified using a high MFI threshold, similar to the approach used with KCl treatment. PABPN1 puncta with an MFI greater than 400 were only observed in A16 cells (Fig. 1E). The fluorescence intensity of these puncta remained unchanged after KCl treatment, confirming that they represent insoluble protein.

To assess aggregate structure in the inducible cell model, we used electron microscopy. In A16 myonuclei, we observed electron-lucent areas (Fig. 1F) that resembled those seen in an OPMD muscle biopsy (Supplementary Fig. 2). These areas contained short fibrils in the muscle cell model (Fig. 1G) and longer fibrils in the muscle biopsy (Supplementary Fig. 2). This suggests that PABPN1 aggregates in A16 cells have characteristics similar to those in OPMD muscle aggregates.

Intranuclear inclusions containing p62 have recently been proposed as a histopathological marker for OPMD [41]. To evaluate the relevance of our cell model for OPMD, we performed co-immunohistochemistry using p62 and FLAG antibodies in A16-FLAG cells. After 5 days of doxycycline treatment, only partial overlap was observed between A16-FLAG and p62 signals, whereas after 7 days, complete overlap was indicated by yellow puncta (Fig. 1H). No FLAG or p62 nuclear puncta were detected in vehicle-treated cultures (Fig. 1H). These results suggest that p62 inclusions in OPMD are associated with PABPN1 nuclear aggregates as secondary events. Overall, the aggregation observed in the A16 muscle cell model closely resembles nuclear aggregates in OPMD, supporting its suitability for studying the molecular mechanisms driven by PABPN1 nuclear aggregation in OPMD.

Muscle cells (myoblasts) undergo differentiation through fusion (Fig. 1I). The resulting differentiated cells are multinucleated and express myosin heavy chain (MyHC) (Fig. 1J). The fusion index, which is measured by the fraction of nuclei in cells that express MyHC, is a quantitative, cell-based readout for muscle cell differentiation [27]. In Dox-treated cell cultures, the fusion index was only around 40% (Fig. 1K), implying that in differentiation conditions, A16 cell culture contains higher proportion of unfused cells compared with fused cells. Omics studies have revealed major molecular differences between differentiated and undifferentiated muscle cells [42, 43]. To minimize these differences, we examined the molecular mechanisms driven by PABPN1 aggregates in proliferating cultures.

### Profiles of bulk mRNAs and proteins distinguish between subcellular fractions

**Figure 2.**
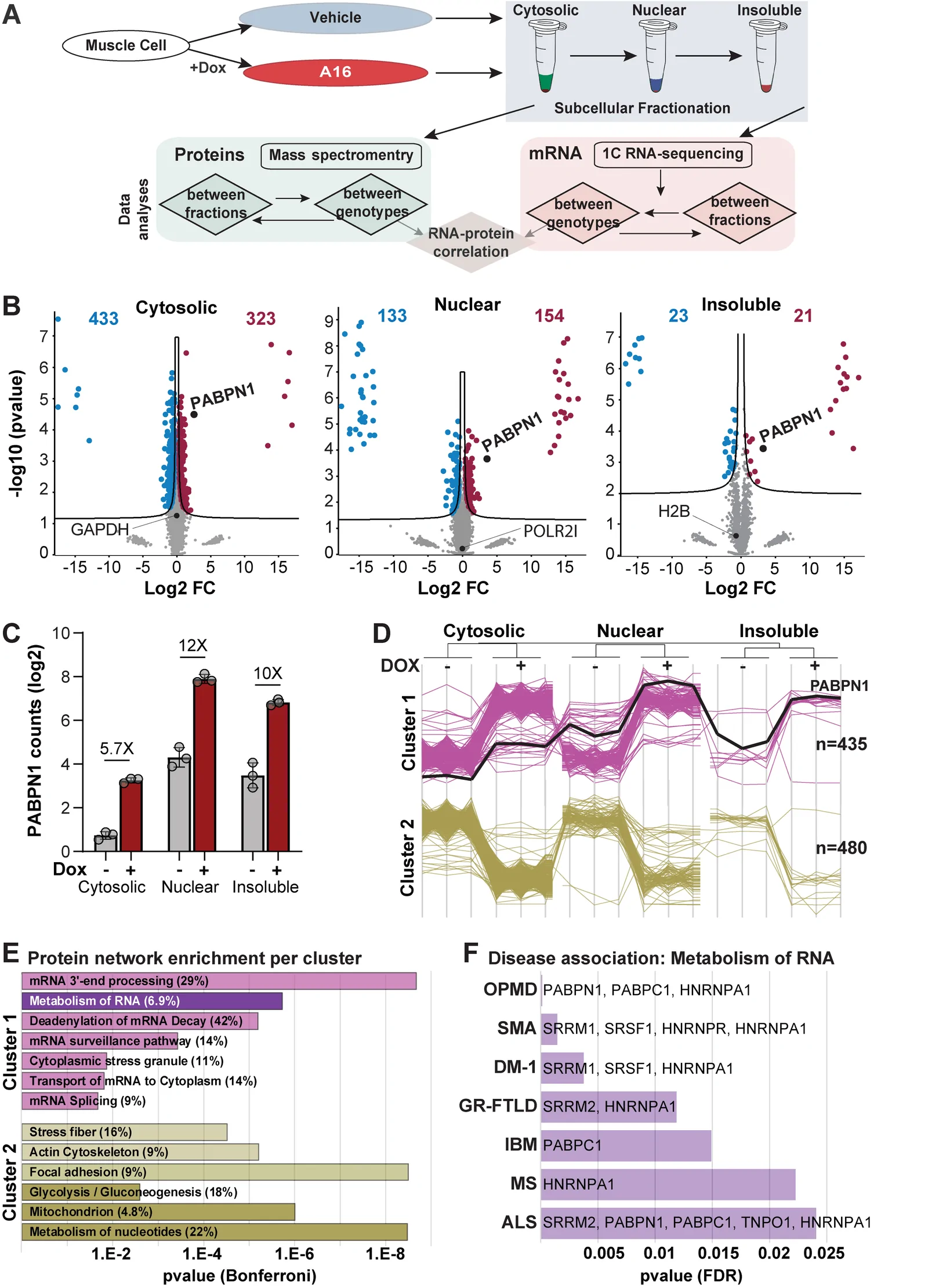
**Subcellular fractionation and analysis of the A16 proteome**. (**A**) A summary of the main fractionation, data generation steps of the A16 proteome and transcriptome analysis. A detailed flowchart of data generation and analysis is found in Figure S3. (**B**) Volcano plots the differential protein abundance in the cytosolic, nuclear, and insoluble fractions. The black line indicates the 5% FDR significance threshold. Higher abundance in A16 is shown in red and lower abundance is shown in blue. Depicted are PABPN1 and GAPDH, POLR2I and H2B, signatures for each cell fraction. (**C**) Bar graph of PABPN1 counts (log2). The average fold change is shown per fraction. (**D**) Clustering plot of differentially abundant proteins in the cytosolic, nuclear, and insoluble fractions. The black line represents PABPN1. The number of proteins per cluster is indicated. (**E**) A bar graph of the pathway enrichment analysis per cluster, the x-axis shows the Bonferroni adjusted p-value. The percentage of A16-enriched proteins is given in parentheses. Functionally related pathways are highlighted with a similar shade. The complete enrichment data can be found in Table S4. The p-value (Bonferroni) was calculated from our proteome as background. (**F**) Protein-disease correlation of the 'Metabolism of RNA' group, p-value is on the x-axis; the associated proteins are labeled. OPMD: oculopharyngeal muscular dystrophy, SMA: spinal muscular atrophy, DM-1: myotonic dystrophy type 1, GR-FTLD: granulin-related frontotemporal lobar degeneration, IBM: Inclusion body myositis, MS: Multiple sclerosis, ALS: Amyotrophic lateral sclerosis).

To evaluate the impact of PABPN1 aggregates on cellular and molecular processes, we performed sequential subcellular fractionation, separating the cells into cytosolic, nuclear, and insoluble fractions (Fig. 2A). Bulk RNA and proteins were isolated from each fraction. Western blot analysis revealed PABPN1 accumulation in the nuclear fraction and A16 in the insoluble fraction; GAPDH was only detected in the cytosolic fraction (Supplementary Fig. 3A). The size distribution of bulk RNA differed between the insoluble and nuclear or cytoplasmic fractions (Supplementary Fig. 3B). We analyzed proteins by mass spectrometry (MS) and examined mRNA profiles using poly(A) 3′-UTR RNA sequencing.

To develop an analysis pipeline to study the effects of A16 on protein and mRNA profiles, we first compared global differences in protein abundance and mRNA accumulation among the three fractions (Fig. 2A and Supplementary Fig. 3C). Significant differences in protein abundance and RNA read counts were observed (Supplementary Fig. 4A). Principal component analysis (PCA) revealed that variation among fractions exceeded variation among genotypes (Supplementary Fig. 4C and 4D). The cytosolic proteome was approximately 10% larger than the nuclear proteome and approximately 33% larger than the insoluble proteome (Supplementary Fig. 4A). The mRNA library size was largest in the cytosolic fraction, nearly 10 times larger than the nuclear fraction and 100 times larger than the insoluble fraction (Supplementary Fig. 4B). Since equal amounts of total RNA were used for library preparation, the small size of the insoluble library suggests that either poly(A) RNA constitutes only ~1% of total RNA in this fraction, or insoluble RNA is poorly suited to protocols optimized for nuclear and cytoplasmic mRNA.

Differential expression analysis revealed significant differences in protein abundance among the fractions. Ninety-four percent of proteins were significantly localized to the nuclear or cytosolic fractions, while 71% were localized to the nuclear or insoluble fractions (Supplementary Fig. 4E). The largest differences occurred between the cytosolic and insoluble fractions. Each fraction was characterized by distinct protein groups: autophagy-related proteins (ATGs) in the cytosolic fraction, centromere proteins (CENPs) in the nuclear fraction, and histone proteins (H2s) in the insoluble fraction. Pearson's correlation analysis revealed a stronger correlation between the nuclear and insoluble protein profiles (Supplementary Fig. 4F). This finding is consistent with the fractionation sequence and suggests that most insoluble proteins originate from the nucleus. Based on these findings, we examined the effect of PABPN1 aggregates on the proteome within each fraction.

Contrary to the proteomic data, mRNA profiles showed 42% similarity between the cytosolic and nuclear fractions. Nearly all of the mRNA detected in the insoluble fraction was of nuclear origin (Supplementary Fig. 4G). The correlation of mRNA abundance was higher between the cytosolic and nuclear fractions than with the insoluble fraction (Supplementary Fig. 4H). These findings confirm that mRNA is largely shared between the nuclear and cytosolic fractions, while the insoluble fraction contains a distinct set. Therefore, we restricted the analysis of A16’s effects on mRNA expression patterns to the nuclear and cytosolic fractions.

### The A16 proteome is enriched by regulators of RNA metabolism

To examine the effect of A16-PABPN1 on the proteome, an analysis was carried out per fraction, considering significance at p<0.05 and correcting for multiple comparisons. Higher PABPN1 levels were found in all fractions (Fig. 2B), consistent with the Western blot (Supplementary Fig. 2A), and GAPDH levels remained unchanged in the cytosolic fraction. The PABPN1 fold change was higher in the nuclear and insoluble fractions than in the cytosolic fraction (12x, 10x, and 5.7x, respectively; see Fig. 2C). Notably, the highest PABPN1 fold change was in the nuclear and insoluble fractions, while the most prominent changes in protein abundance were in the cytosolic fraction. The abundance of 13.5% of the proteins in the cytosolic fraction was affected, compared to 5.9% and 1.3% in the nuclear and insoluble fractions, respectively (Supplementary Fig. 5A).

We then explored a pattern across the A16 proteome (n = 1,087 proteins) using Euclidean clustering. Two major clusters were identified (Fig. 2D and Supplementary Fig. 5B). Proteins in Cluster 1 showed higher abundance across the fractions, similar to PABPN1, whereas proteins in Cluster 2 displayed the opposite pattern (Supplementary Fig. 5B). In cluster 2, proteins were enriched primarily in metabolic processes such as nucleic acid biosynthesis, mitochondrial processes, and glucose metabolism. These proteins were also enriched in pathways that regulate cell structure, including focal adhesion, the actin cytoskeleton, and stress fibers (Fig. 2E and Supplementary Table 4).

**Figure 3.**
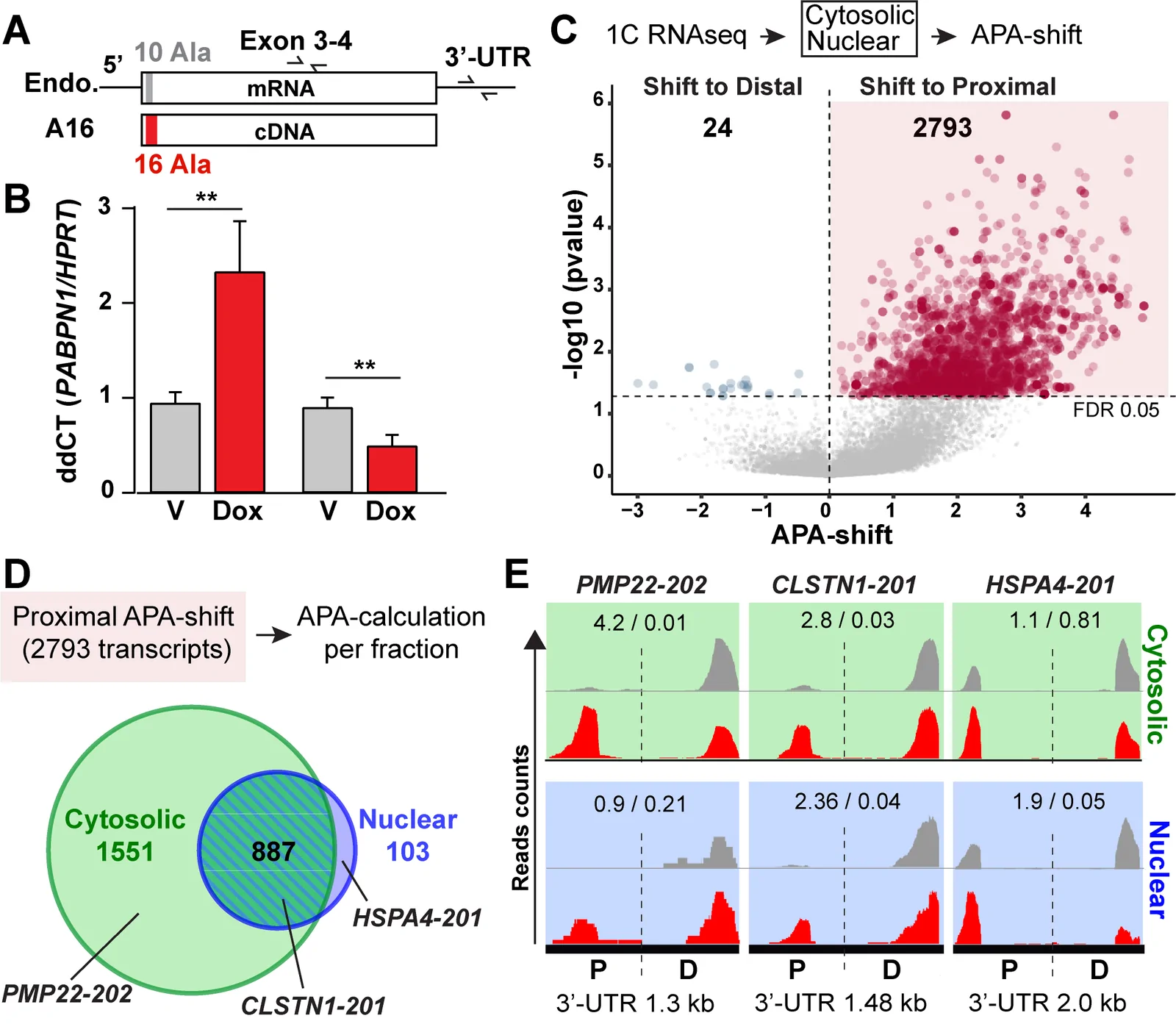
**APA-shift in A16 cells correlates with A16 overexpression and endogenous downregulation**. (**A**) Schematic representation of the wild-type (Ala10) and expanded (Ala16) transgenes and the endogenous PABPN1 primers at the 3'-UTR distinguishing the endogenous from the transgene cDNA. The position of the primers is indicated by arrows. Primers to exons 3-4 amplify both the transgene and endogenous PABPN1, and primers to the 3'-UTR amplify only the endogenous transcript. (**B**) Bar graph of RT-qPCR analysis of PABPN1 levels detected with exon3-4 or 3’-UTR primer set, normalized to HPRT in vehicle-treated (V) and Dox-induced cells. The mean and standard deviation are from N=3. Statistical significance was assessed by one-way ANOVA; p<0.01 is indicated by **. (**C**) Top: A summary of APA-shift analysis in cytoplasmic and nuclear fractions. Bottom: The volcano plot shows the APA-shift fold change between vehicle-treated and Dox-induced cells. Transcripts with significant proximal APA-shift are in red (N=2793) and distal APA-shift in blue (N=24). The dashed line indicates the 5% FDR significance threshold. (**D**) A summary of APA-shift analysis per fraction: the APA-shift was recalculated per fraction for the 2793 transcripts in 4C. Venn diagram shows APA-shift transcripts per fraction and the overlap between fractions. Transcript examples are depicted. (**E**) IGV plots of reads count at the 3’-UTR, the vehicle-treated is in gray, and the Dox-induced in red. The Y-axis shows the reads count per distal or proximal region 3’-UTR on the X-axis. IGV plots are from a representative replicate. Per gene the APA-shift value/p-value (FDR) are denoted per fraction. The length of the 3'-UTR is in kb. PMP22-202 is significant in the cytosolic fraction only, HSPA4-201 is significant in the nuclear fraction only, and CLSTN1-201 is significant in the cytosolic and nuclear fractions. P: proximal, D: distal.

**Figure 4.**
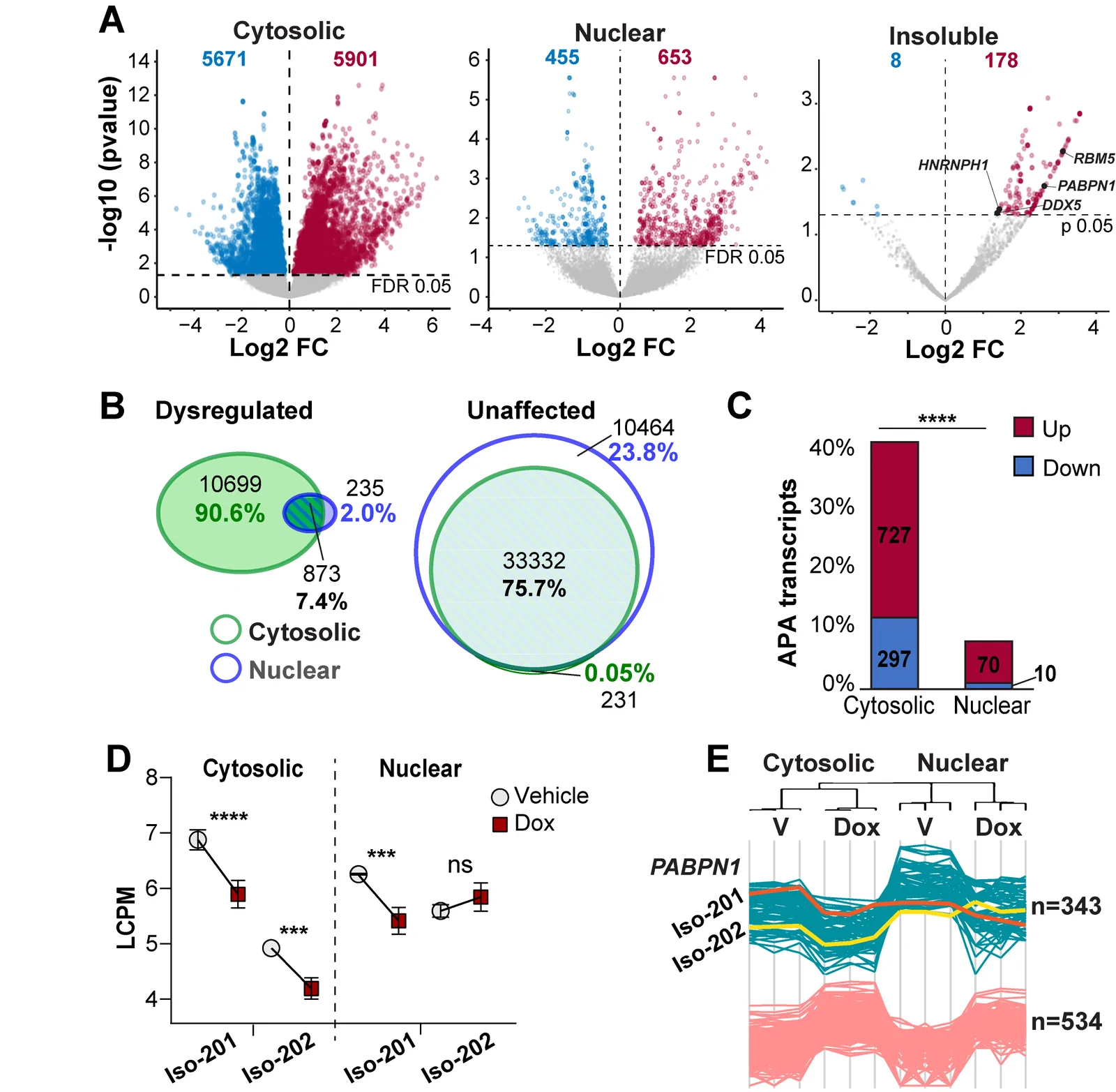
**The A16 transcriptome across cell fractions**. (**A**) Volcano plots of differential transcript abundance in the cytosolic and nuclear fractions. The dashed line indicates the 0.05 FDR and p-value significance threshold. Upregulation in the Dox-induced condition is shown in red, and downregulation in blue shows the number of down- or upregulated transcripts. Examples of upregulated transcripts in the insoluble fraction are highlighted. (**B**) Venn diagrams of the overlap of transcripts in the cytosolic (in green) and nuclear (in blue) fractions in the A16 dysregulated or in the unaffected transcripts. The numbers of transcripts and the percentage are denoted. (**C**) Bar graph of down- or up-regulated transcripts with APA in cytosolic or nuclear fractions. The numbers of transcripts is denoted. The percentage is calculated from the total APA-shift. Statistical significance was calculated using the chi-square test; p<0.0001 is indicated with ****. (**D**) PABPN1 isoform expression changes in cytosolic and nuclear fractions. Full-length PABPN1 isoforms (201, 202) are indicated. The mean and standard deviation are from N=3. Statistical significance was assessed by two-way ANOVA; p<0.001 and p<0.0001 are indicated by *** and ****, respectively. (**E**) A clustering plot of A16 dysregulated mRNAs with APA-shift in cytosolic and nuclear fractions. PABPN1 isoforms are indicated. The number of mRNAs per cluster is indicated.

Mitochondria, stress fibers, and the actin cytoskeleton play fundamental roles in muscle cell differentiation. Reduced expression levels of these proteins are consistent with the reduced fusion index observed in the A16 cell culture (Fig. 1K). In the cytosol, 100 dysregulated muscle proteins (Supplementary Table 3) were significantly enriched in sarcomeric structures, such as the I-band and Z-disc, as well as in proteins involved in cell adhesion, including those in anchoring junctions and focal adhesions (Supplementary Fig. 6A). Sarcomeric gene expression is regulated by the MYO1D transcription factor, which was expressed at 50% of normal levels in the A16 nuclear fraction (Supplementary Table 3). No significant enrichment of muscle proteins was found in the nuclear fraction (N = 30) (Supplementary Fig. 6A). Reduced expression of MYOD1 and sarcomeric proteins is consistent with reduced myogenesis in A16 cells and suggests that PABPN1 affects differentiation capacity.

**Figure 5.**
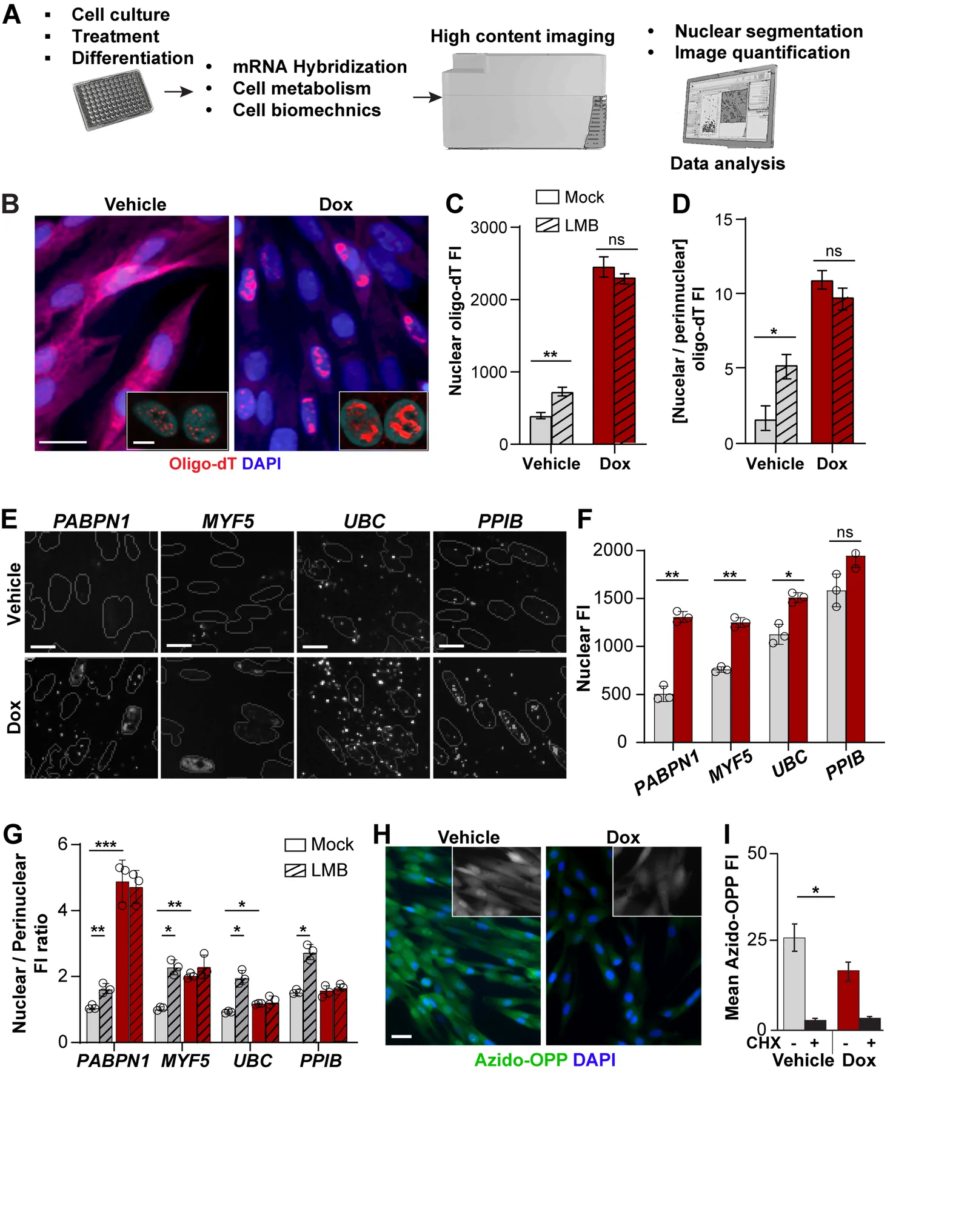
**High throughput analysis of cell function in A16 cell culture**. (**A**) Schematic of high-throughput cell-based analysis: cells were seeded in a 96-well plate and treated with Dox for 3-4 days, followed by staining for RNA hybridization or cellular metabolism, imaging, and image quantification. The nuclear region (N) and the perinuclear region (P-N) are depicted, and the fluorescence intensity in both regions were used for calculation. (**B**) Images show oligo-dT-Cy5 (red) hybridization in vehicle-treated and Dox-induced cells. The scale bar is 40 μm. Nuclear counterstaining is shown in blue. Corresponding confocal images are shown in the insets. The scale bar is 15μm. (**C**) Oligo-dT intensity in vehicle-treated and Dox-induced cells in mock or LMB (37.5 nm)-treated cell cultures. Bar graph of nuclear oligo-dT fluorescence intensity (FI). (**D**) Bar graph of nuclear to perinuclear [N/P-N] oligo-dT FI. (**E**) Images of RNAscope staining of four genes (PABPN1, MYF5, UBC, PPIB.) in vehicle-treated and Dox-induced cell cultures. The nuclear segmentation is outlined. The scale bar is 10 μm. (**F**) Bar graph show the mean nuclear spots fluorescence intensity (FI) per nucleus object for *PABPN1, MYF5, UBC*, and *PPIB*. Each circle represents one biological replicate, >1500 cells per replicate. G. Bar graph shows the average nuclear to perinuclear [N/P-N] of single mRNA spot for *PABPN1, MYF5, UBC*, and *PPIB*. Each circle represents one biological replicate, >1500 cells per replicate. (**H**) Images show Azido-OPP (green) and nuclear counterstaining in vehicle-treated or Dox-induced cell cultures. The scale bar is 50 μm. (**I**) Bar graph of Azido-OPP FI in vehicle-treated and Dox-induced cell cultures. Cycloheximide (CHX) was used as a control. In all experiments average and standard deviations are from N=3 biological replicates. Statistical significance was assessed by two-way ANOVA: p<0.05 and p<0.01 are indicated by * and **, respectively. Means and standard deviations are from N=3 biological replicates.

In contrast, proteins in cluster 1 were only enriched for RNA-binding protein gene sets (Fig. 2E). These included A16-dysregulated RNA-binding proteins (RBPs) involved in mRNA 3′ untranslated region (3′UTR) binding, translation regulation, single-stranded RNA binding, RNA helicase activity, and poly(A) binding (Supplementary Fig. 6B). Several of these proteins exhibited fraction-specific fold changes. For instance, IGF2BP1 recruits transcripts to cytoplasmic protein-RNA complexes. Additionally, MRPL44 and MRPS7 are mitochondrial ribosomal proteins (Supplementary Fig. 6C). These findings suggest that A16-PABPN1 aggregation impairs RNA processing and metabolism in both nuclear and cytosolic RNA fractions.

To predict the involvement of A16-RBP proteins in other pathologies, we examined gene-disease associations. Six diseases were significantly enriched, and all were neuromuscular: OPMD, SMA, myotonic dystrophy type 1 (DM-1), GR-FTLD, IBM, MS, and ALS (Fig. 2F). Five of these diseases are categorized as protein aggregation: OPMD, DM-1, GR-FTLD, IBM, and ALS. These results suggest that aberrant PABPN1 expression affects the expression of multiple RPBs, thereby disrupting multiple layers of RNA metabolism.

### PABPN1 aggregation leads to reduced PABPN1 mRNA and APA impacting mRNA subcellular localization

Aggregation of expanded PABPN1 has been suggested to associate with reduced PABPN1 transcript levels [27]. Using two primer sets, we detected reduced levels of endogenous PABPN1 in A16 cells. One primer set amplified both the Ala16-PABPN1 transgene and the endogenous PABPN1 exon region, while the other primer set amplified only the endogenous transcript 3′ UTR region (Fig. 3A). These results confirm that our cell model exhibits a hallmark of OPMD and suggest that overexpressing Ala16-PABPN1 reduces overall PABPN1 expression.

To evaluate the impact of PABPN1 aggregation on its molecular function, we conducted RNA sequencing and calculated APA-shift values, which quantify genome-wide alterations in alternative polyadenylation (APA) usage [27]. We analyzed APA-shift in the cytosolic and nuclear fractions, excluding the insoluble fraction due to low transcript counts. Since no differences were anticipated between the two fractions, they were initially examined together (Fig. 3C). Most transcripts shifted to proximal polyadenylation sites (Fig. 3C), which is consistent with the results from the A17.1 mouse model [19, 27]. The vast majority (96%) of the APA transcripts were cytosolic (Fig. 3D). The APA-shift calculation was demonstrated for three examples (Fig. 3E): PMP22-202 (APA shift: 4.2; FDR: 0.015) and HSPA4-201 (APA shift: 1.92; FDR: 0.045), which were found only in the cytosol and nucleus, respectively, as well as CLSTN1-201, which was significant in both fractions (FDR: 0.029 and 0.042). The high proportion of APA transcripts in the cytosol is consistent with previous findings that short 3′ UTRs are more stable due to less efficient degradation [44]. Overall, aggregation in our model reduces PABPN1 expression, thereby impairing its molecular function.

Differential expression analysis revealed that the strongest effect of Ala16-PABPN1 occurred in the cytosolic fraction. In this fraction, 25% of transcripts (n = 11,572) were dysregulated, compared to only 2.5% (n = 1,108) in the nuclear fraction (Fig. 4A). The insoluble fraction was excluded due to low read counts. Nuclear export analysis showed that, of the transcripts unaffected by Ala16, 0.05% were cytosol-specific, 23.8% were nucleus-specific, and 75% were shared. In contrast, only 7.4% of the dysregulated transcripts overlapped between the fractions; 90.6% were cytosol-specific (Fig. 4B). These results suggest that, while most unaffected transcripts undergo normal export, the expression of Ala16-PABPN1 reduces mRNA turnover, leading to cytoplasmic accumulation. This is consistent with the enrichment of RNA decay proteins in the A16 proteome.

Using the 1C protocol to assess mRNA dynamics in situ, we examined APA transcript localization. Dysregulated APA-shift transcripts were more frequent in the cytosol (42%) than in the nucleus (8%) (Fig. 4C). Most of these transcripts were upregulated, suggesting cytosolic accumulation.

Endogenous PABPN1 isoforms (iso-201 and iso-202) were significantly downregulated in the cytosol (Fig. 4D), which is consistent with the results of the qRT-PCR (Fig. 3B). Clustering the A16 dysregulated transcripts with APA shift identified two groups: a "blue" cluster with expression pattern acroos fractions similar to PABPN1 and a "red" cluster (534 transcripts) with opposite pattern (Fig. 4E).

**Figure 6.**
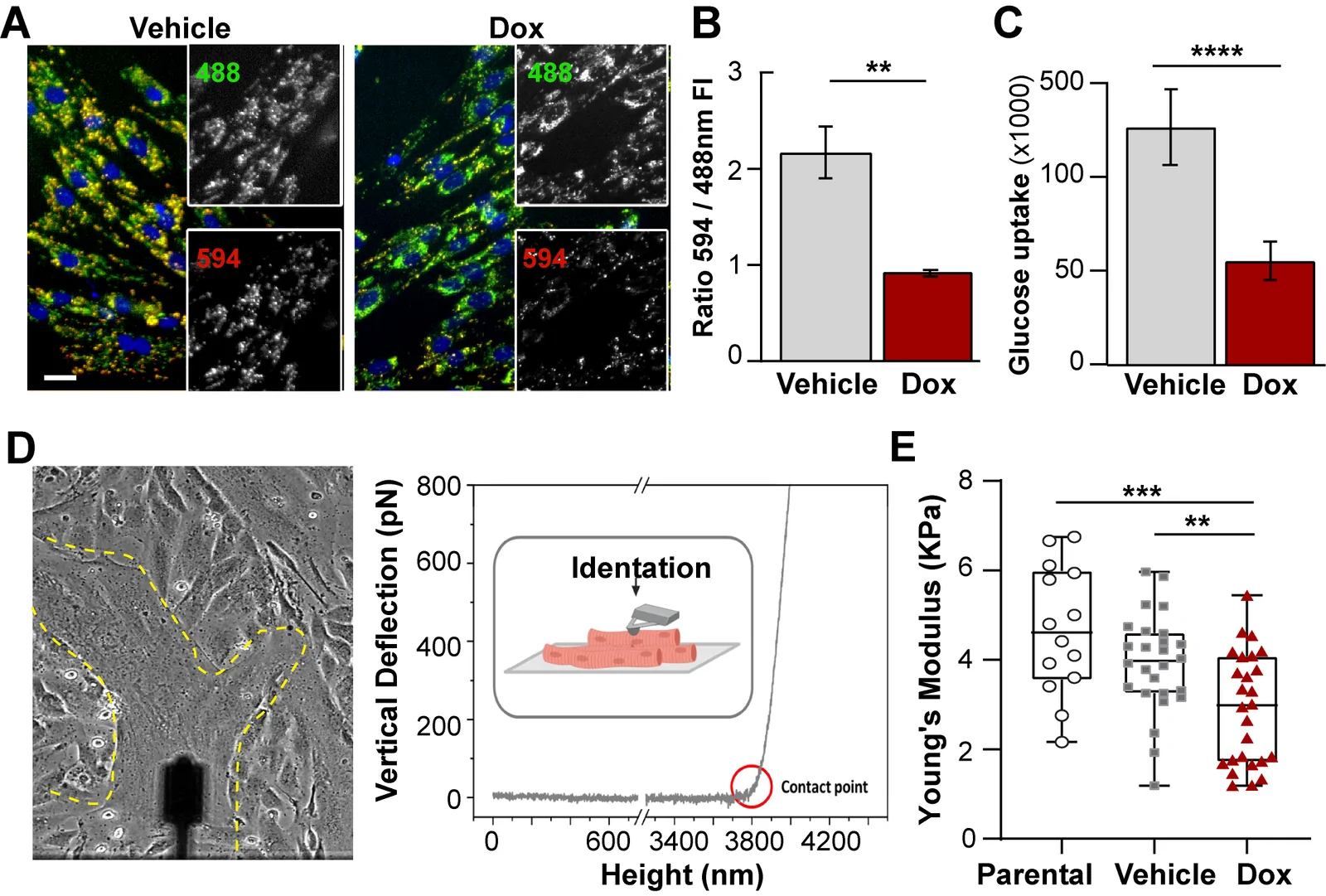
**A16 impaired muscle cell metabolism, differentiation and biomechanics**. (**A**) Images show JC1 fluorescence in vehicle-treated or Dox-induced cell cultures. The monomers are stained green, the activated J-aggregates are stained red, and the image per fluorophore is shown in the grayscale inset. The nuclear counterstain is shown in blue. The scale bar is 40 μm. (**B**) Bar graph of 594/488nm FI ratio in vehicle-treated and Dox-induced cell cultures. The mean and standard deviation are from N=3 biological replicates. (**C**) Bar graph of glucose uptake assay in vehicle-treated and Dox-induced cell cultures. The mean and standard deviation are from N=4 biological replicates. (**D**) CellHesion image of differentiated cell culture (left). A multinucleated cell is marked by a yellow dashed line. The cantilever is black. (Right) A typical CellHesion measurement curve showing the contact point of the cantilever with the cell. The inset shows a schematic of the cantilever's contact with the cell membrane. (**E**) Membrane stiffness (Young's modulus) in parental, vehicle-treated, and Dox-induced multinucleated cells. Every dot represents a myofiber. Statistical significance was assessed by one-way ANOVA: p<0.01; p<0.005 or p<0.001 are indicated by **, ***, or ****, respectively.

Our paired RNA-protein study design allowed us to investigate whether A16-PABPN1's effect on transcript folding affects protein folding directly. We examined the correlation between transcripts in the cytosolic fraction and their corresponding proteins in both the cytosolic and nuclear fractions. Linear regression analysis of the entire proteome revealed weak direct relationships between protein and transcript fold-change in both fractions (Supplementary Fig. 7). Only 0.5% and 0.2% of proteins in the cytosolic and nuclear fractions, respectively, were predicted to exhibit linear correlation. For significantly dysregulated transcripts and proteins, the correlation in the nuclear fraction was insignificant (F-test = 0.316). Only 1.6% of deregulated nuclear proteins directly correlated with transcript fold changes (Supplementary Fig. 7). In the cytosolic fraction, the linear regression model suggested a weak positive correlation; 9.9% of protein fold changes directly correlated with transcript fold changes (Supplementary Fig. 7). The accumulation of nuclear and cytosolic proteins is differentially affected by A16-PABPN1.

### Poly(A) nuclear accumulation in A16 cells is associated with reduced nuclear export, translation efficiency, and cell metabolism

To investigate the impact of pathways significantly affected in A16 cells, we used high-content imaging and image quantification (Fig. 5A). Using the oligo-dT-Cy5 in situ hybridization protocol, we studied the effect of A16-PABPN1 mRNA localization in the nucleus and cytosol. Oligo-dT-Cy5 nuclear puncta were only observed in A16 cells (Fig. 5B). These Oligo-dT-Cy5 puncta colocalize with A16-YFP aggregates [25]. The mRNA signal in Dox-treated cells was 2.5 times higher than in vehicle-treated cells (Fig. 5C). The nuclear-to-perinuclear ratio increased fivefold in A16 cells compared to vehicle-treated cells (Fig. 5D). These results suggest impaired nuclear exports and/or trafficking of poly(A) RNA. We evaluated nuclear mRNA export using leptomycin B (LMB), which inhibits exportin-1 and the export of RNAs containing an NES sequence [45]. In LMB-treated cells, the oligo-dT-Cy5 signal in the nucleus was more intense than in mock-treated cells (Supplementary Fig. 9). We found a twofold increase in the nuclear oligo-dT signal in LMB-treated vehicle cells, but it remained unchanged in Dox-treated cells (Fig. 5C and 5D). To confirm the nuclear accumulation of mRNA in A16 cells, we employed single-molecule RNA in situ hybridization. Based on RNA sequencing results, we selected the upregulated genes UBC, MYF5, and PABPN1 as well as PPIB as an unchanged control (Fig. 5E; RNA expression levels are shown in Supplementary Fig. 8). The PABPN1 probe detects both Ala16-PABPN1 and endogenous PABPN1. Quantification of RNA single-molecule puncta revealed a higher signal in nuclear regions for PABPN1, MYF5, and UBC in A16 cells, while the signal for PPIB remained unchanged (Fig. 5F). The higher nuclear-to-perinuclear ratio of PABPN1, MYF5, and UBC in A16 cells is consistent with the RNA sequencing analysis (Fig. 5G and 5H). LMB treatment resulted in a higher puncta signal in vehicle-treated cells but not in A16 cells (Fig. 4G), which is consistent with the results of the oligo-dT in situ hybridization (Fig. 5D). These results suggest that A16 cells have impaired nuclear export of poly(A) RNA.

Because APA affects translation efficiency, we measured global translation efficiency using azido-OPP (Fig. 5H). The azido-OPP fluorescence signal decreased with cycloheximide treatment, and the OPP signal was lower in A16 cell cultures than in cultures treated with the vehicle (Fig. 5I). Taken together, these results suggest that A16 cell mRNA nuclear entrapment is associated with reduced poly(A) RNA nuclear export and translational efficiency.

Among the A16-affected cytosolic pathways (Fig. 2E), we verified the effect of A16-PABPN1 on mitochondrial activity and glucose uptake. Mitochondrial activity was significantly lower in A16 cells than in vehicle-treated cells (Fig. 6A and 6B). Similarly, glucose uptake was significantly lower in A16 cells than in vehicle-treated cells (Fig. 6C). Additionally, the A16 proteome was enriched for pathways affecting muscle cell differentiation and cell biomechanics, including the cytoskeleton, focal adhesion, and stress fibers (Fig. 2E). Single-cell measurements of cell force in differentiated cells revealed decreased cell stiffness in A16 cells (Fig. 6D and 6E). These results suggest that reduced expression of cytoskeletal and focal adhesion proteins negatively impacts cell differentiation and biomechanics.

## DISCUSSION

Protein aggregates are a hallmark of aging and age-related diseases. These aggregates disrupt protein homeostasis networks via a feed-forward regulatory loop affecting multiple cellular processes [46, 47]. Pathogenic protein aggregates can be either cytosolic, as in ALS or Parkinson's disease, or nuclear, as in OPMD. Protein clearance pathways differ between the nuclear and cytoplasmic regions [48]. This underscores the need for studying disease mechanisms in subcellular regions. Here, we provide insights into the molecular networks and cellular functions driven by nuclear aggregates produced by the pathogenic form of PABPN1. Unlike other studies that elucidate how protein aggregates affect cell function in unaffected cell types [49, 50], we modeled protein aggregation in human muscle cells by overexpressing expanded PABPN1 twofold. In comparison, the A17.1 mouse model of OPMD was generated using overexpression levels approximately tenfold higher than in our inducible cell system [51, 52]. Conversely, only limited PABPN1 aggregation was observed in cells with lower overexpression (1.5 fold, [53]), or in a knock-in mouse model of OPMD [54, 55]. These findings suggest that modeling PABPN1 aggregation requires at least twofold overexpression.

Recent studies have highlighted the specificity of proteostasis control and protein aggregation to different cell types. The aggregates in our human muscle cells exhibit several features in common, including resistance to KCl treatment, presence of fibrils in the electron-lucent region, and nuclear sequestration of p62. We also demonstrate that the molecular function of PABPN1, specifically APA suppression, is impaired in this cell model. Taken together, the cellular and histological descriptions of our cell model demonstrate its suitability for studying the effects of PABPN1 aggregates on muscle cell function.

PABPN1 is predominantly nuclear, and in OPMD, the insoluble aggregates are also nuclear. However, the A16 proteome was mostly affected in the cytoplasmic fraction. Pathways associated with A16-PABPN1 expression levels were primarily enriched for RNA metabolic pathways. Some RBPs are prone to aggregation, which is consistent with their predicted association with neuromuscular diseases and protein aggregation disorders, such as DM-1, GR-FTLD, IBM, and ALS. PABPN1 has been implicated in the nuclear export of mRNA [21], and we demonstrate here that nuclear export is impaired in A16 cells. Additionally, the mRNA surveillance pathway was significantly enriched in the A16 proteome, which is consistent with the accumulation and entrapment of poly(A) RNA in A16 nuclei. The A16-enriched cytoplasmic pathways include proteins involved in cell metabolism energy, and cell structure, that are mostly downregulated in A16 cells. We demonstrated reduced mitochondrial activity, and glycolysis, as well as reduced myogenesis in A16 cell cultures. These two energy production pathways have been associated with many neuromuscular diseases, including protein aggregation disorders [56, 57]. Disruption of protein networks that shape the cell structure is also common in models of protein aggregation disorders [58-60]. Our study demonstrates a significant effect on cell biomechanics that could translate to muscle weakness in OPMD patients. Importantly, the same cytosolic pathways were found to be affected in muscle models with reduced PABPN1 levels [61]. Reduced expression of PABPN1 expression affects the expression cytoskeletal proteins, muscle cell fusion, and cell biomechanics [62]. These findings suggest that reduced PABPN1 levels or PABPN1 aggregation result in similar cellular dysfunction. Our results are consistent with the growing body of evidence indicating a strong link between cellular metabolism and mechanics [63].

Co-localization experiments in PABPN1 nuclear aggregates revealed the sequestration of various proteins, including proteostasis regulators. Here, we demonstrate that p62 is also sequestered in PABPN1 aggregates. This finding is consistent with p62 accumulation in OPMD and may explain the reduced autophagy observed in OPMD models [51, 64]. Although the expression of genes involved in the ubiquitin-proteasome system (UPS) and autophagy have been associated with OPMD [65], and proteasomal activity is impaired in OPMD models [6, 66], we did not observe significant dysregulation of protein networks that regulate protein homeostasis. We found the co-localization of p62 with PABPN1 aggregates subsequent to mRNA sequestration. This suggests that UPS and autophagy dysregulation are not initially affected by OPMD. Since the UPS and autophagy dysfunction are associated with aging [46], it is possible that prolonged culture time with PABPN1 nuclear aggregates may affect protein levels of the UPS and autophagy protein levels.

The nuclear accumulation of mRNA puncta in A16 cells is consistent with previous studies of OPMD models [67], and with a co-localization with YFP-PABPN1 nuclear aggregates [25]. Therefore, we expected to find high mRNA levels in the insoluble fraction. However, in contrary to the oligo-dT hybridization results, the library size in the insoluble fraction was 100-fold or 10-fold lower than in the soluble cytoplasmic or nuclear fractions, respectively. It is possible that our RNA isolation protocol is not optimal for the insoluble fraction. Nevertheless, the detected transcripts were enriched in A16 insoluble fraction, which coincides with the oligo(dT) signal in PABPN1 aggregates. The enrichment of mRNA with TDP-43 aggregates was reported in cytoplasmic stress granules [68]. he sequestration of mRNA in RBP aggregates suggests disruption of normal RNA metabolism and transport processes, which could contribute to disease pathology [69].

Here, we demonstrate that the molecular function of PABPN1, which is the regulation of APA, is impaired in the A16 cell model. APA is present in cell cultures with PABPN1 downregulation [19]. APA is also present in the A17.1 mouse model and, to a lesser extent, in OPMD models, both of which have reduced PABPN1 mRNA levels [27]. Therefore, we propose that OPMD pathology is driven by PABPN1 loss of function, caused by reduced mRNA and soluble protein levels, and PABPN1 aggregation, which depletes soluble nuclear proteins. Loss of PABPN1 function results in APA dysregulation and aberrant nuclear mRNA export. PABPN1 aggregates sequester nuclear proteins and bulk mRNA, including multiple RBPs. Consistent with this model, the loss of TDP-43 function due to aggregation has been proposed as a disease mechanism in ALS, which is associated with nuclear accumulation and reduced export [70], which is associated with nuclear accumulation and reduced nuclear export [71]. However, it is unclear how PABPN1 aggregates lead to reduced PABPN1 levels. However, a previous study suggested that PABPN1 regulates its own expression through RNA editing. Overexpression of PABPN1 results in the accumulation of unspliced PABPN1 transcripts, reducing endogenous PABPN1 mRNA levels [72]. Here, we demonstrate that PABPN1 mRNA is sequestered in the nuclei of A16 cells, resulting in decreased cytoplasmic mRNA levels and reduced protein levels.

Taken together, our data suggest that known pathogenic RNA-binding proteins may be associated with PABPN1 aggregates, which affect cell biomechanics. Based on these findings, we propose that OPMD pathology involves disruption of mRNA metabolism at multiple regulatory levels and subcellular regions regulated by multiple RBPs. Our results show that alternative polyadenylation (APA) is associated with RNA sequestration within insoluble nuclear aggregates, affecting nuclear export and translation efficiency. Furthermore, we propose that OPMD pathology is caused by the loss of PABPN1 function driven by PABPN1 aggregation. A better understanding of PABPN1's role in the cytosol and its intracellular dynamics could facilitate the development of therapeutic strategies for OPMD and other RBP protein aggregation diseases.

## Supplementary Materials

The Supplementary data can be found online at: www.aginganddisease.org/EN/10.14336/AD.2025.0699.



## Data Availability

The raw mass spectrometry proteomics data have been deposited to the ProteomeXchange Consortium via the PRIDE partner repository with the dataset identifier PXD055515. The sequencing data generated in this study have been deposited in the Gene Expression Omnibus (GEO) under accession number GSE277571. The data are publicly available at www.ncbi.nlm.nih.gov/geo/query/acc.cgi?acc=GSE277571. Further metadata and related files can be found in the accompanying submission.
