# Supplementary material for: Nuclear Protein Aggregates Disrupt RNA Processing and Alter Biomechanics in a Muscle Cell Model of OPMD
Source: Aging Dis. 2026 Aug 18;17(5):2583–603. doi: 10.14336/AD.2025.0699 (PMC13437119; doi:10.14336/AD.2025.0699)
Supplement: Supplementary file 1 [file AD-17-5-2583-s.pdf]

SUPPLEMENTARY DATA

Nuclear Protein Aggregates Disrupt RNA Processing and Alter Biomechanics in a Muscle Cell Model of OPMD

Milad Shademan, Sarah Flannery, Erik Bos, Tom M.J. Evers, Vahid Sheikhhassani, Alireza Mashaghi, Benno Kusters, Baziel van Engelen, Thomas H. Sharp, Roman Fischer, Benedikt M. Kessler, Vered Raz

© 2025. Shademan M et al. Published online at http://www.aginganddisease.org/EN/10.14336/AD.2025.0699

SUPPLEMENTARY DATA

SUPPLEMENTARY TABLES

Supplementary Table 1. List of antibodies

**Primary antibodies**					
**Target**	**Dilution**		**Host**	**Supplier**	**Cat. Number / Reference**
	**Immunofluorescence**	**Western blot**			
GAPDH	-	1:5000	Mouse	Invitrogen	MA5-15738
PABPN1	1:1000	1:4000	Rabbit	Homemade	DOI: 10.1016/j.ajpath.2024.12.009
FLAG	1:1000		Mouse	Sigma-Aldrich	F1804
P62	1:250		Rabbit	Cell Signaling	SQSTM1/p62
SC35	1:250		Mouse	Sigma-Aldrich	S4045
Histon2B (H2B)	-	1:1000	Rabbit	Proteintech	15857-1-AP
MyHC	1:250	-	Mouse	DSHB	MF20

**Secondary antibodies**			
**Target**	**Fluorophore**	**Dilution**	**Supplier**
Anti-Rabbit	Cy5	1:2000	ThermoFisher Scientific
Anti-Mouse	Alexa488	1:2000	ThermoFisher Scientific
Anti-Mouse	Alexa594	1:2000	ThermoFisher Scientific
WB anti-Rb/M	800/680	1:10000	IRDye 800CW or IRDye 680RD (LI-COR)

Supplementary Table 2. List of primers for RT-qPCR

**Target**	**Forward 5' – 3'**	**Reverse 5' – 3'**
HPRT1	TGGTCAGGCAGTATAATCCAAAGA	TCAAATCCAACAAAGTCTGGCTTA
PABPN1_exon 3-4	ATGTTGGCAATGTGGACTATG	ACACGGTTGACTGAACCACA
PABPN1_3'-UTR	ATGGACACGTCTCAACTGCG	ACGGAGGGAAGGTAACAAGC

Supplementary Table 4. Proteome cluster enrichment analysis

**Cluster 1**				
**Category**	Term	Count	Fold Enrichment	Bonferroni
**REACTOME_PATHWAY**	R-HSA-72187~mRNA 3'-end processing	17	10.65	2.1E-09
**REACTOME_PATHWAY**	R-HSA-72203~Processing of Capped Intron-Containing Pre-mRNA	30	3.89	7.1E-07
**REACTOME_PATHWAY**	R-HSA-8953854~Metabolism of RNA	50	2.55	1.9E-06
**GOTERM_CC_DIRECT**	GO:1990904~ribonucleoprotein complex	21	5.02	4.4E-06
**REACTOME_PATHWAY**	R-HSA-429947~Deadenylation of mRNA	10	15.40	6.5E-06
**KEGG_PATHWAY**	hsa03015:mRNA surveillance pathway	14	5.44	3.7E-04
**GOTERM_CC_DIRECT**	GO:0016607~nuclear speck	26	3.02	1.1E-03
**REACTOME_PATHWAY**	R-HSA-72163~mRNA Splicing - Major Pathway	19	3.39	1.3E-02
**GOTERM_CC_DIRECT**	GO:0010494~cytoplasmic stress granule	11	5.64	1.4E-02
**REACTOME_PATHWAY**	R-HSA-72202~Transport of Mature Transcript to Cytoplasm	12	5.28	1.5E-02
**GOTERM_CC_DIRECT**	GO:0071013~catalytic step 2 spliceosome	11	5.47	1.8E-02
**REACTOME_PATHWAY**	R-HSA-72172~mRNA Splicing	19	3.27	2.1E-02

**Cluster 2**				
**Category**	Term	Count	Fold Enrichment	Bonferroni
**REACTOME_PATHWAY**	R-HSA-15869~Metabolism of nucleotides	22	7.04	3.1E-09
**GOTERM_CC_DIRECT**	GO:0005925~focal adhesion	37	3.93	3.2E-09

© 2025. Shademan M et al. Published online at http://www.aginganddisease.org/EN/10.14336/AD.2025.0699

**GOTERM_CC_DIRECT**	GO:1904813~ficolin-1-rich granule lumen	19	7.02	1.1E-07
**GOTERM_CC_DIRECT**	Mitochondrion (4.8%)	69	2.17	9.9E-07
**GOTERM_CC_DIRECT**	GO:0015629~actin cytoskeleton	25	4.11	6.2E-06
**KEGG_PATHWAY**	hsa00051:Fructose and mannose metabolism	11	10.00	2.1E-05
**GOTERM_CC_DIRECT**	GO:0001725~stress fiber	14	7.27	3.1E-05
**KEGG_PATHWAY**	hsa01250:Biosynthesis of nucleotide sugars	10	8.35	5.7E-04
**KEGG_PATHWAY**	hsa00010:Glycolysis / Gluconeogenesis	12	5.54	2.5E-03
**GOTERM_CC_DIRECT**	GO:0034774~secretory granule lumen	13	5.18	4.2E-03
**KEGG_PATHWAY**	hsa00480:Glutathione metabolism	11	5.86	4.2E-03
**KEGG_PATHWAY**	hsa01200:Carbon metabolism	15	4.00	6.1E-03
**KEGG_PATHWAY**	hsa00520:Amino sugar and nucleotide sugar metabolism	10	6.18	7.9E-03
**GOTERM_CC_DIRECT**	GO:0035578~azurophil granule lumen	11	5.59	1.4E-02
**GOTERM_CC_DIRECT**	GO:0043202~lysosomal lumen	11	5.19	2.6E-02

Table S3: (A) Proteome differential analysis; (B) A16-associated muscle proteins and RBPs
Table S5: APA shift analysis
Table S6: mRNA differential expression analysis
Table S7: mRNA cluster enrichment analysis

The Tables S3, S5, and S7 can be requested from the corresponding editor.

© 2025. Shademan M et al. Published online at http://www.aginganddisease.org/EN/10.14336/AD.2025.0699

SUPPLEMENTARY DATA

SUPPLEMENTARY FIGURES

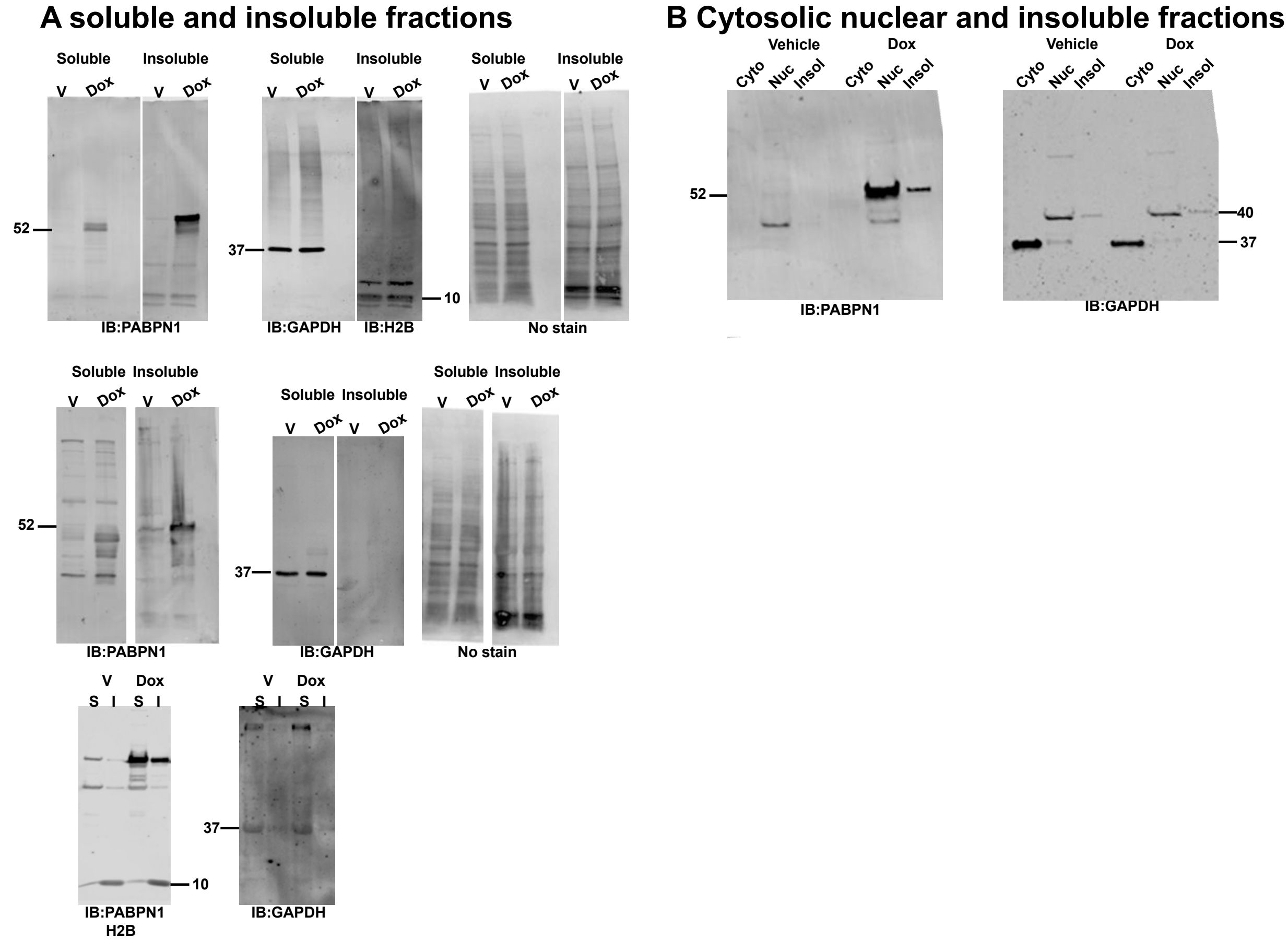

Supplementary Figure 1. Full images' western blots. A. Soluble vs insoluble fractions; **B.** Subcellular fractionation Proteins that are detected by the antibodies are indicated. No-stain is a bulk protein staining. H2B is an insoluble protein, and GapDH a soluble cytosolic protein. Protein molecular weight is denoted.

© 2025. Shademan M et al. Published online at http://www.aginganddisease.org/EN/10.14336/AD.2025.0699

SUPPLEMENTARY DATA

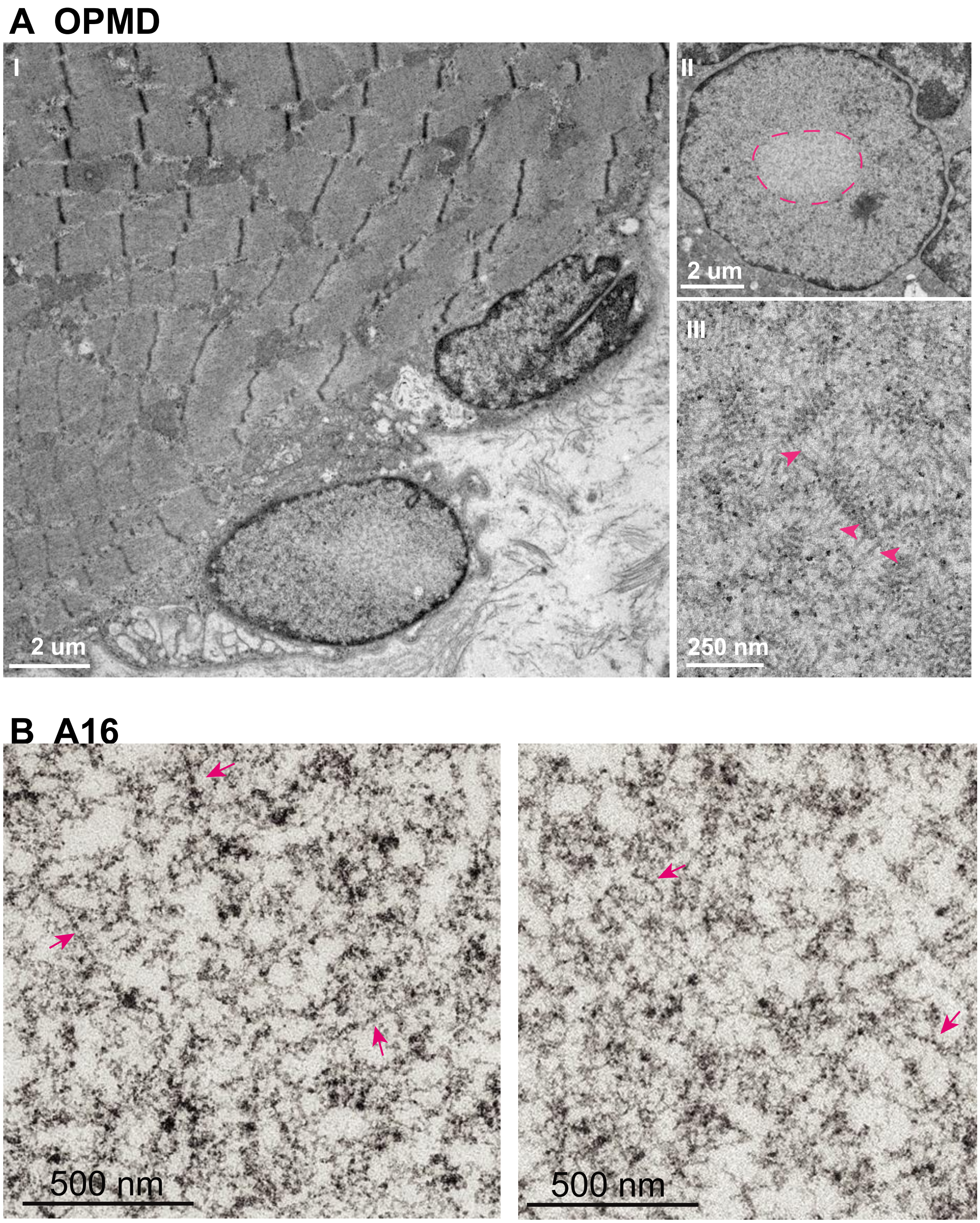

Supplementary Figure 2. Electron microscopy details A. muscle from an OPMD patient**. I.** Image of myofibers and myonuclei. **II.** A myonucleus with a lucent area (dashed line). **III.** A magnified area of the lucent region showing. **B. A16 nuclei.** Pink fibrils are denoted with pink arrows.

© 2025. Shademan M et al. Published online at http://www.aginganddisease.org/EN/10.14336/AD.2025.0699

SUPPLEMENTARY DATA

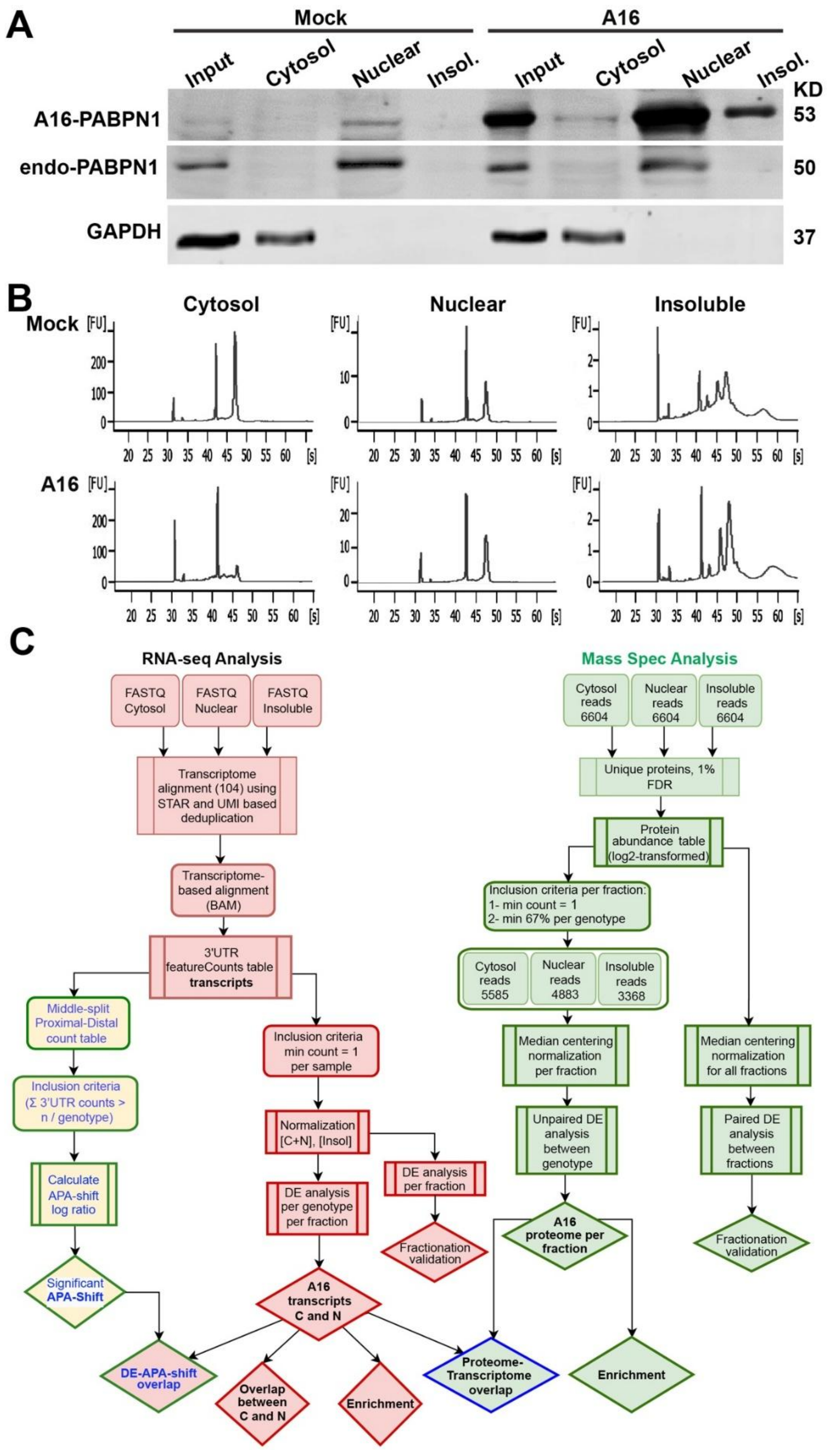

Supplementary Figure 3. Subcellular fractionation A. Western blot analysis: A16-PABPN1; endogenous PABPN1 (endo-) and GAPDH in vehicle-treated and Dox-treated cells in cytoplasmic, nuclear, and insoluble fractions. Input is before fractionation. **B.** Bioanalyzer RNA output showing cytosolic, nuclear, and insoluble fractions. **C.** Detailed flowchart of A16 subcellular fractions and data analysis. The green color is the pipeline used for protein data analysis. Red is used for RNA and yellow is used for APA calculation.

© 2025. Shademan M et al. Published online at http://www.aginganddisease.org/EN/10.14336/AD.2025.0699

SUPPLEMENTARY DATA

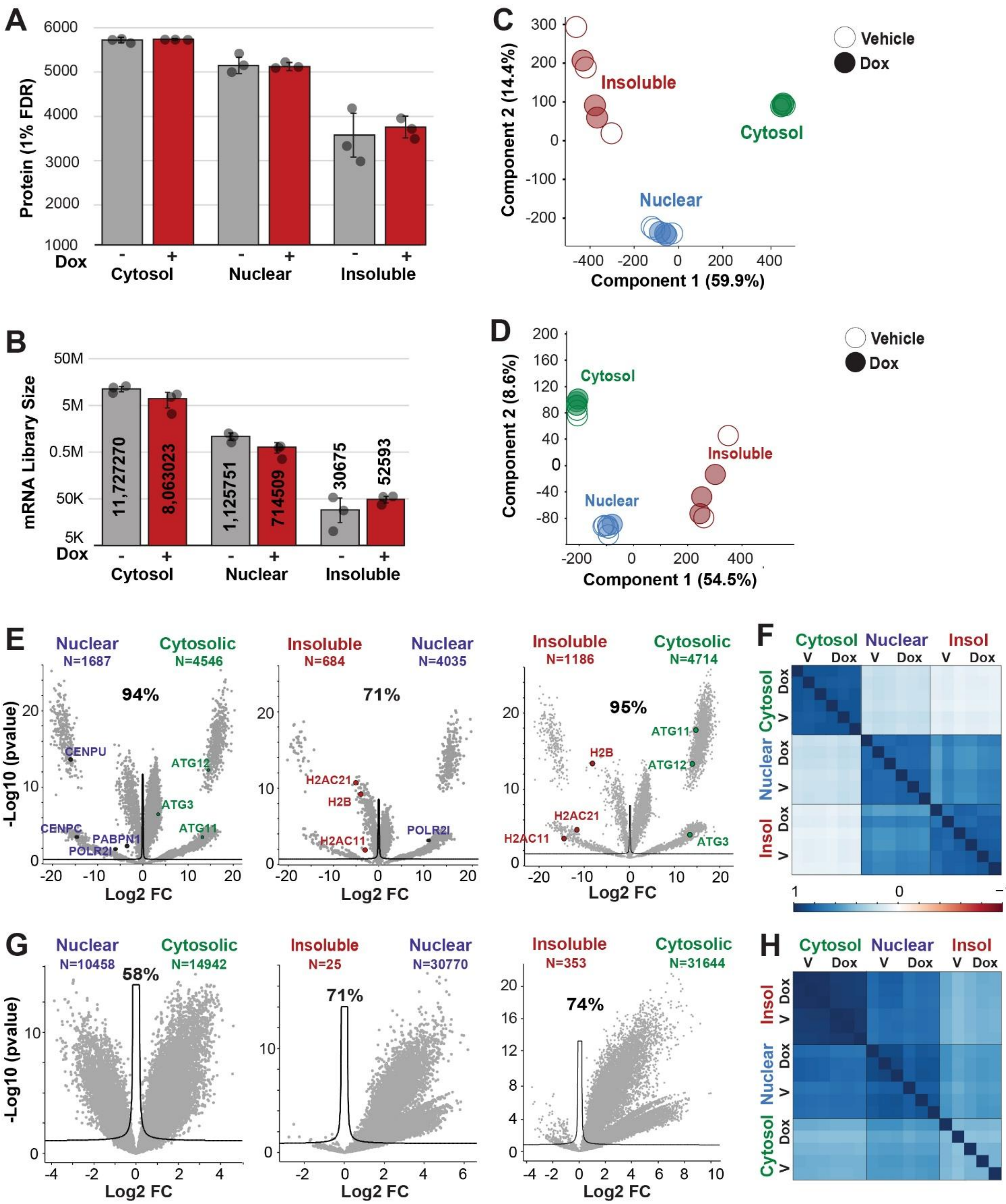

Supplementary Figure 4. QC RNA sequencing and mass spectrometry and analysis between fractions. A. Bar graph of the average protein count per sample after filtering. **B.** Bar graphs show the average library size [=sum of reads (Distal+roximal) at the 3'-UTR] per sample after filtering. The average number of sum reads is denoted in each bar. The mean and standard deviation in A and B are from N=3. For insoluble vehicle-treated mRNA, N=2. **C-D.** Principal component analysis plots of proteins (C) or mRNAs (D) show the main variants

© 2025. Shademan M et al. Published online at http://www.aginganddisease.org/EN/10.14336/AD.2025.0699

and the percentage of variation for component 1 and component 2. **E-F** Protein analysis. **G-H** mRNA analysis. **E** and **G** Volcano plots of differential analysis between cytosolic and nuclear fractions (left), nuclear and insoluble fractions (middle) and cytosolic and insoluble fractions (right). The number (N) of significant proteins or transcripts is indicated per fraction. The percentage of significant proteins or transcripts from the total is shown per analysis. The black line indicates statistical significance ($p<0.05$, FDR). Examples of proteins with distinct subcellular localization are marked as autophagy proteins in the cytosol, centromere binding proteins, POLR2I and PABPN1 in the nuclear fraction, and histones in the insoluble fraction. The encircled protein clusters represent proteins that were detected exclusively in one fraction and not the other. **F.** and **H.** Heatmap correlation matrix between 18 samples. The correlation scale, blue to red, is depicted.

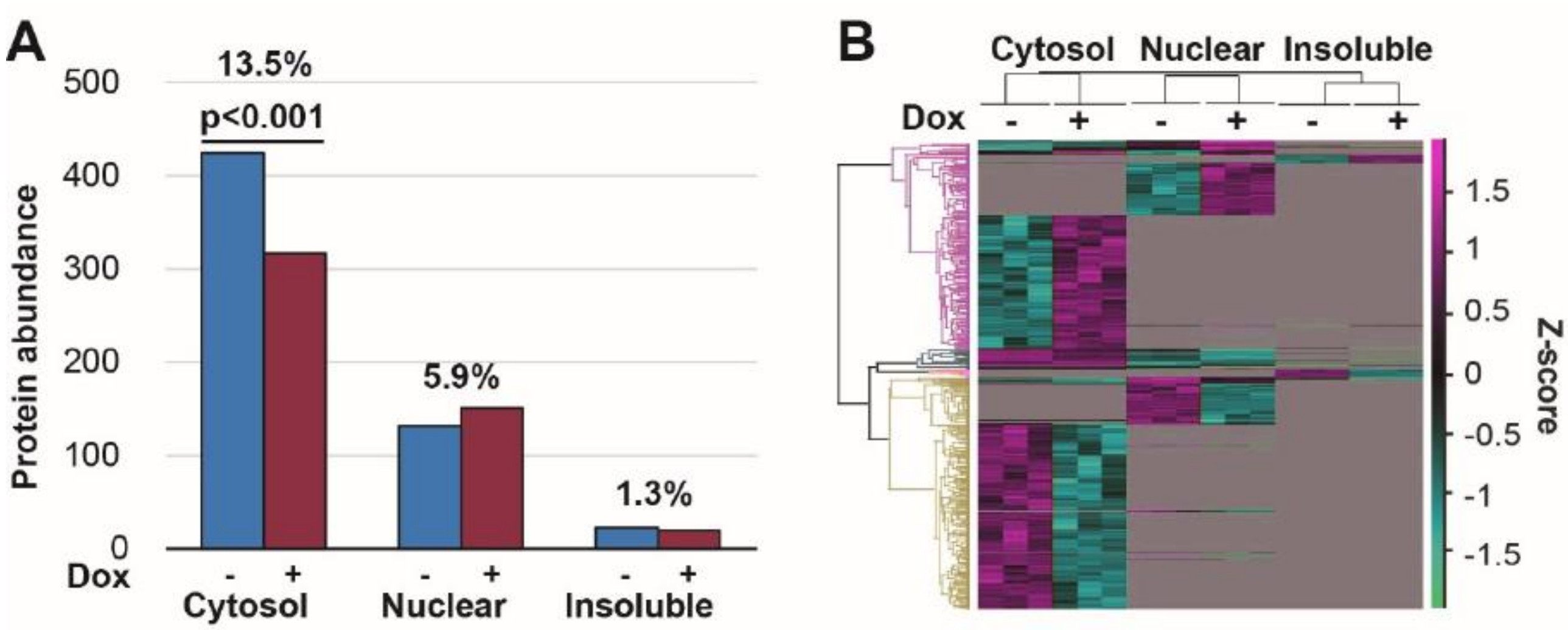

Supplementary Figure 5. The A16 proteome: additional analysis. A. A bar graph of differentially accumulated proteins per fraction. The percentage of differentially accumulated proteins per fraction is derived from the total proteins in the mass spectrometry. A difference between high and low protein abundance was assessed with a chi-squared test. **B.** Clustering of the A16 proteome. Heatmap of significant differential protein abundant in all fractions in vehicle-treated or Dox-induced conditions. Relative expression intensity is indicated on a cyan to purple scale. No value is available for the proteins in the gray area per fraction.

© 2025. Shademan M et al. Published online at http://www.aginganddisease.org/EN/10.14336/AD.2025.0699

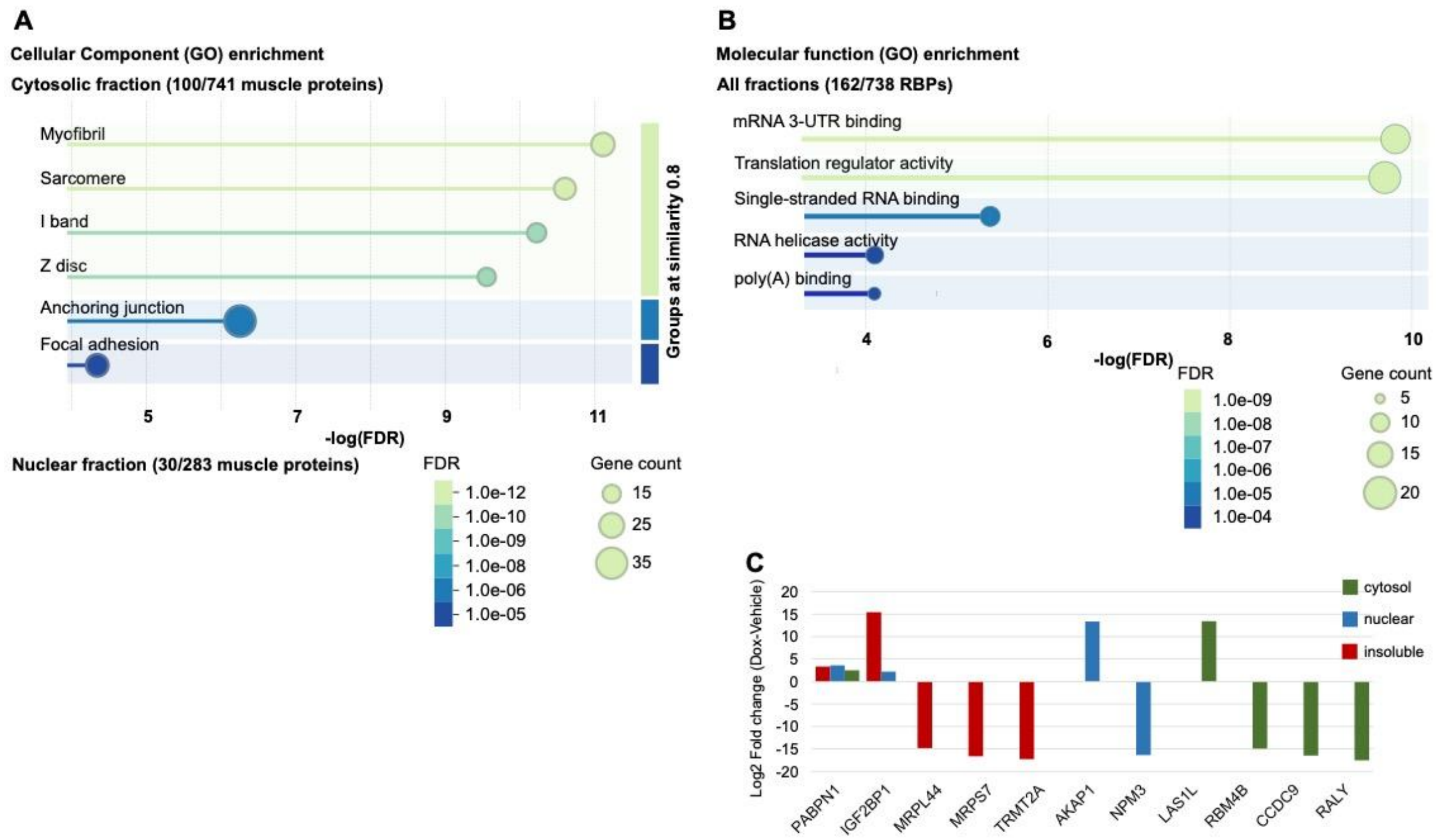

Supplementary Figure 6. Analysis of muscle and RNA binding proteins in the A16 proteome: additional analysis. A. Enrichment analysis of the A16 muscle proteins. The A16 muscle protein list was made by the overlap between muscle protein list (Table S3B) the A16 cytosolic and nuclear proteome. The number of dysregulated proteins and the dysregulated proteins is depicted per fractions. The pvalue is calculated using the proteome in this study as background. **B** and **C.** A16 RNA binding proteins. The A16 RBP protein list was made by the overlap between RBP protein list (Table S3B) the A16 cytosolic and nuclear and insoluble proteome. B. Enrichment analysis of the A16 PRBs proteins. The pvalue is calculated using the proteome in this study as background. The number of dysregulated proteins and the dysregulated proteins is depicted. **C.** Fold change (log2) of the highlighted proteins per fraction.

© 2025. Shademan M et al. Published online at http://www.aginganddisease.org/EN/10.14336/AD.2025.0699

SUPPLEMENTARY DATA

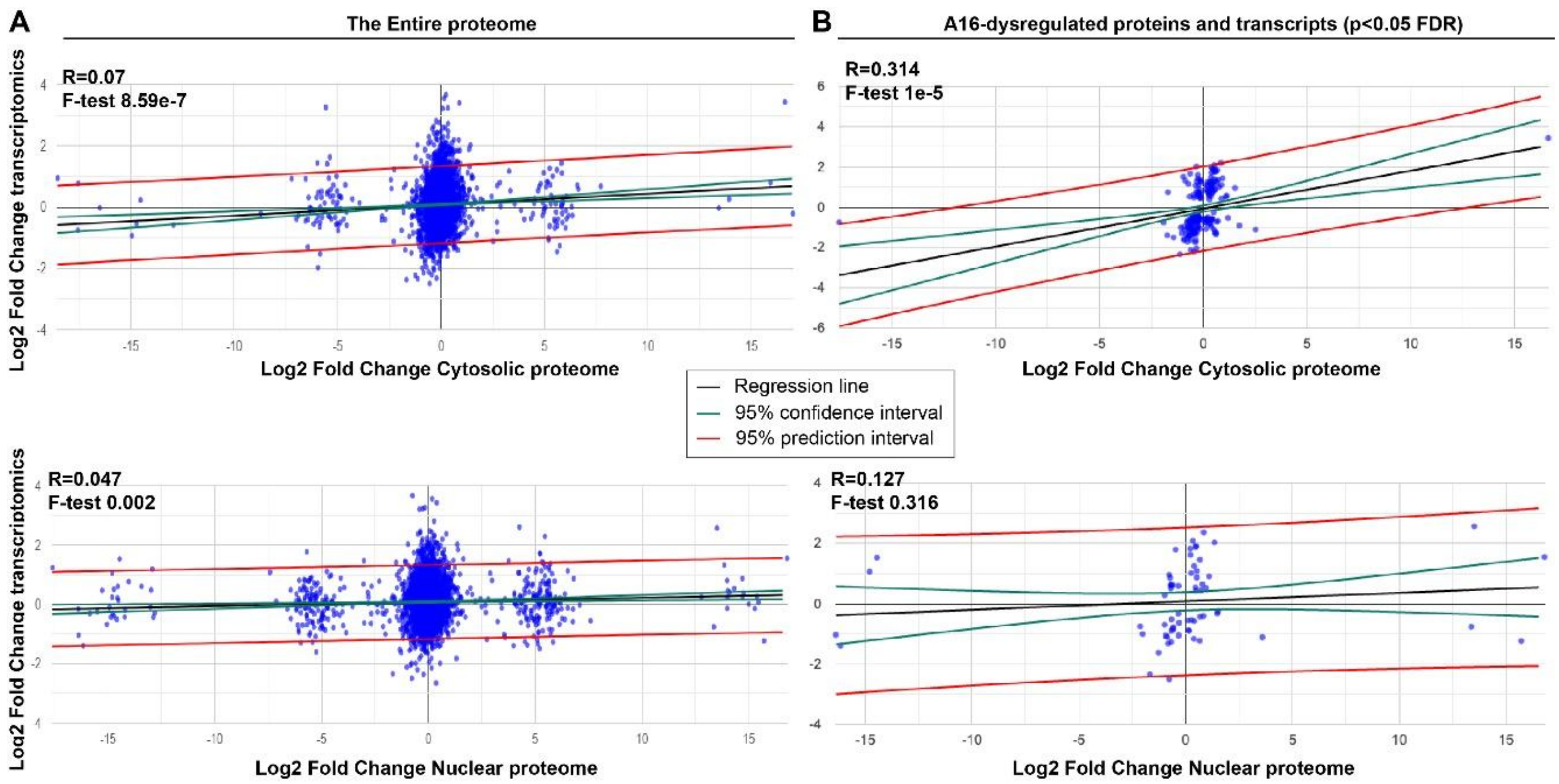

Supplementary Figure 7. A16 Correlation between A16 proteome and transcriptome. A. A linear correlation analysis of the cytosolic transcriptome with the entire cytosolic (upper plot) or nuclear (lower plot) proteome. **B.** A linear correlation analysis of the significant cytosolic transcriptome with the significant cytosolic (upper plot) or nuclear (lower plot) proteome. The regression line is shown in black, the 95% confidence interval in green, and the 95% prediction interval in red. The correlation coefficient (R) and the F-test p-value of the regression lines are shown. The number of up- or down-regulated proteins (and the percentage) correlating with the transcript direction are given.

© 2025. Shademan M et al. Published online at http://www.aginganddisease.org/EN/10.14336/AD.2025.0699

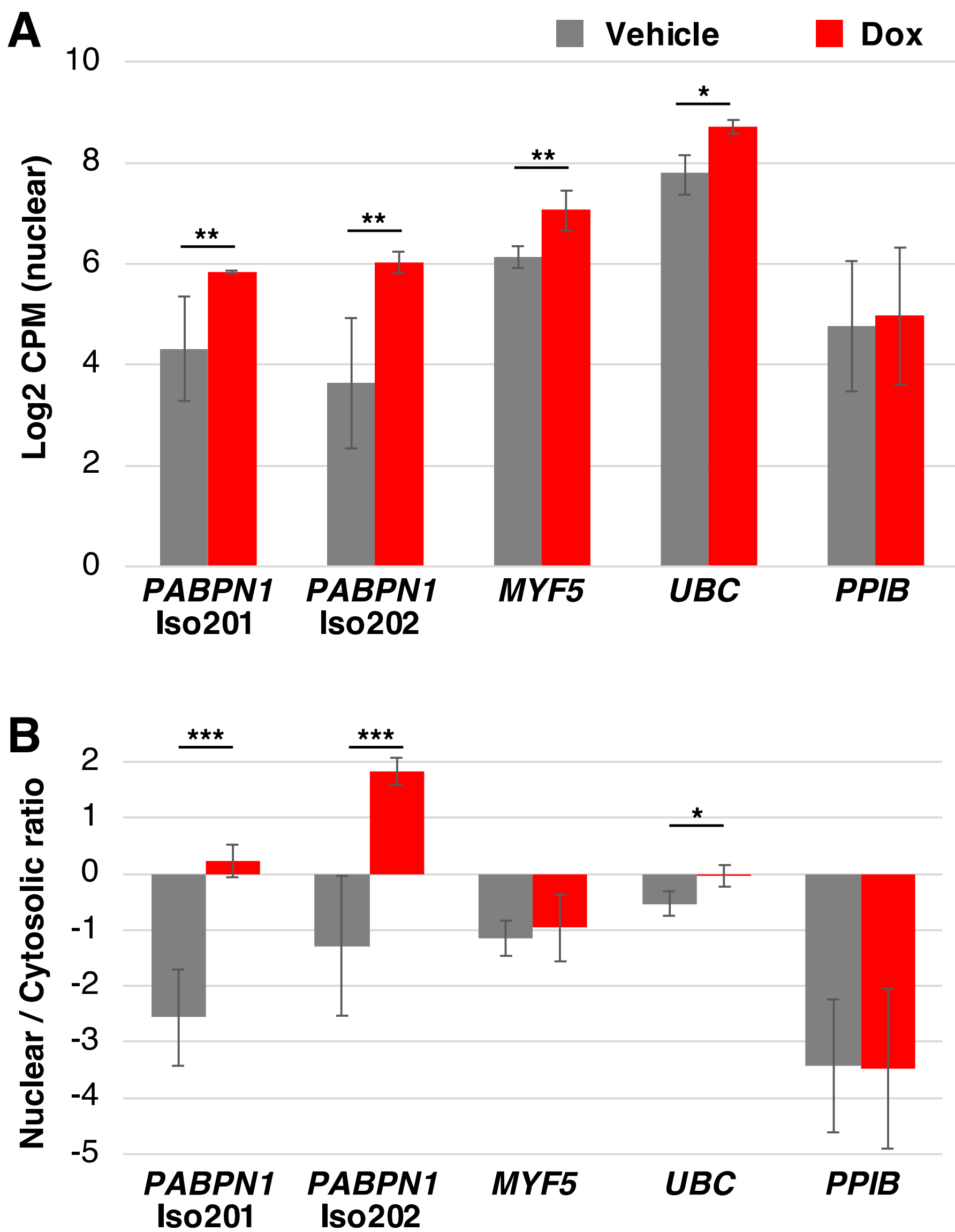

Supplementary Figure 8. RNA-seq analysis of candidate genes in vehicle and Dox treated cells. Additional information for Figure 6F and 6G. A. Transcripts reads (log2 CPM) in nuclear and insoluble fractions. **B.** Nuclear to cytoplasmic Transcripts reads (log2 CPM). The nuclear fraction is as in A. Averages and standard deviations are from N=3. Statistical significance was assessed with the student's t-test. P<0.05 (*); p<0.01 (**); p<0.005 (***).

© 2025. Shademan M et al. Published online at http://www.aginganddisease.org/EN/10.14336/AD.2025.0699

SUPPLEMENTARY DATA

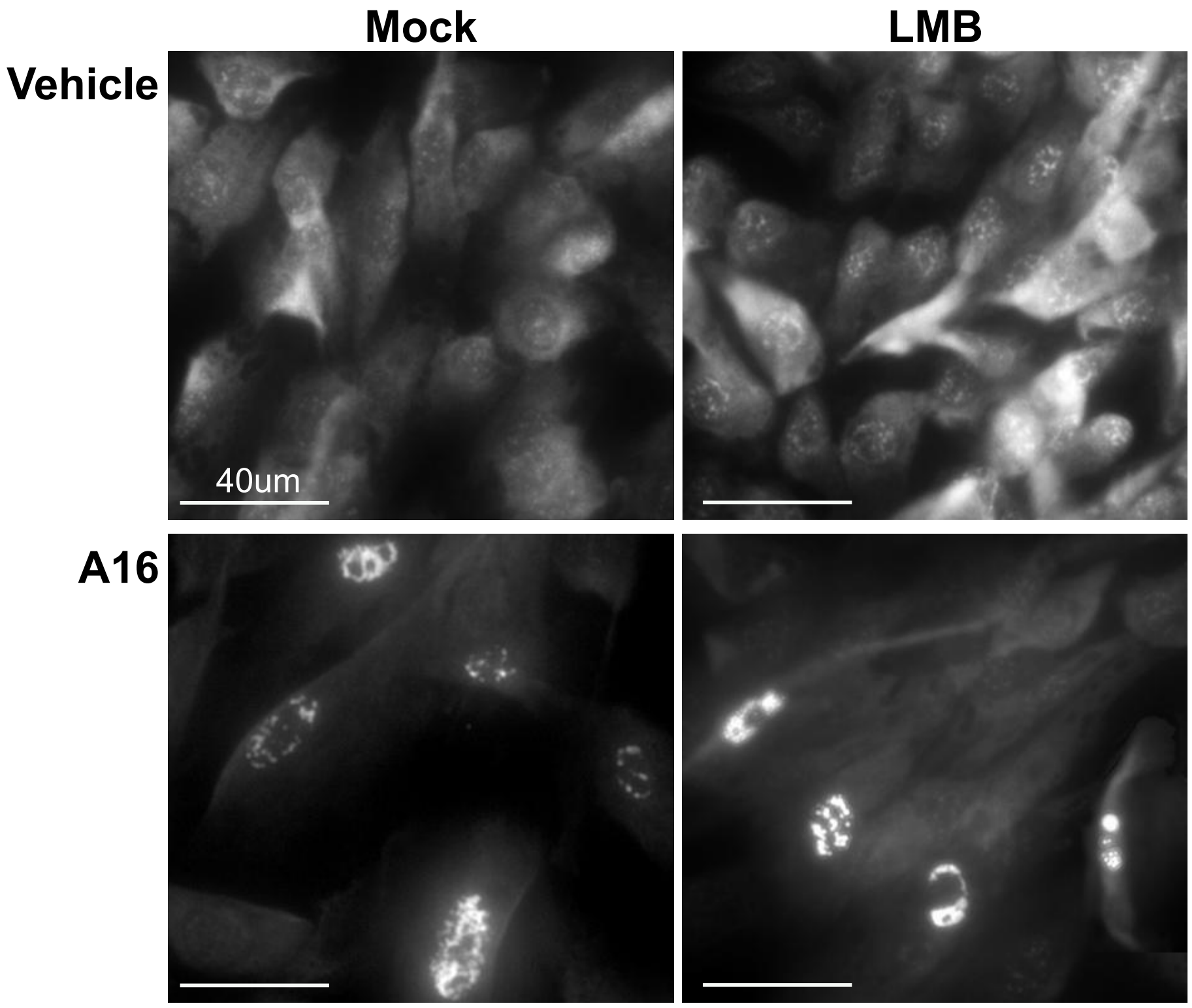

Supplementary Figure 9. Representative images of oligo-dT-Cy5 in situ hybridization in mock or LMB treated vehicles or A16 cells. The scale bar in 40mm.

© 2025. Shademan M et al. Published online at http://www.aginganddisease.org/EN/10.14336/AD.2025.0699
